# The Retrohoming of Linear Group II Intron RNAs in *Drosophila melanogaster* Occurs by Both DNA Ligase 4–Dependent and –Independent Mechanisms

**DOI:** 10.1371/journal.pgen.1002534

**Published:** 2012-02-16

**Authors:** Travis B. White, Alan M. Lambowitz

**Affiliations:** Institute for Cellular and Molecular Biology, Department of Chemistry and Biochemistry and Section of Molecular Genetics and Microbiology, University of Texas at Austin, Austin, Texas, United States of America; Fred Hutchinson Cancer Research Center, United States of America

## Abstract

Mobile group II introns are bacterial retrotransposons that are thought to have invaded early eukaryotes and evolved into introns and retroelements in higher organisms. In bacteria, group II introns typically retrohome via full reverse splicing of an excised intron lariat RNA into a DNA site, where it is reverse transcribed by the intron-encoded protein. Recently, we showed that linear group II intron RNAs, which can result from hydrolytic splicing or debranching of lariat RNAs, can retrohome in eukaryotes by performing only the first step of reverse splicing, ligating their 3′ end to the downstream DNA exon. Reverse transcription then yields an intron cDNA, whose free end is linked to the upstream DNA exon by an error-prone process that yields junctions similar to those formed by non-homologous end joining (NHEJ). Here, by using *Drosophila melanogaster* NHEJ mutants, we show that linear intron RNA retrohoming occurs by major Lig4-dependent and minor Lig4-independent mechanisms, which appear to be related to classical and alternate NHEJ, respectively. The DNA repair polymerase θ plays a crucial role in both pathways. Surprisingly, however, mutations in Ku70, which functions in capping chromosome ends during NHEJ, have only moderate, possibly indirect effects, suggesting that both Lig4 and the alternate end-joining ligase act in some retrohoming events independently of Ku. Another potential Lig4-independent mechanism, reverse transcriptase template switching from the intron RNA to the upstream exon DNA, occurs *in vitro*, but gives junctions differing from the majority *in vivo*. Our results show that group II introns can utilize cellular NHEJ enzymes for retromobility in higher organisms, possibly exploiting mechanisms that contribute to retrotransposition and mitigate DNA damage by resident retrotransposons. Additionally, our results reveal novel activities of group II intron reverse transcriptases, with implications for retrohoming mechanisms and potential biotechnological applications.

## Author Summary

Group II introns are bacterial mobile elements thought to be ancestors of introns and retrotransposons in higher organisms. They consist of a catalytically active intron RNA and an intron-encoded reverse transcriptase, which function together to promote intron integration into new DNA sites in a process called “retrohoming.” In bacteria, retrohoming occurs by the excised intron lariat RNA fully reverse splicing into a DNA site, where it is reverse transcribed, yielding an intron cDNA that is copied directly into the host genome. However, little is known about how group II introns behave in higher organisms. Here, we find that linear group II intron RNAs, which cannot fully reverse splice, retrohome in *Drosophila melanogaster* by attaching themselves to only one end of a DNA site. Reverse transcription then yields an intron cDNA, which is integrated into the recipient DNA by host enzymes that function in non-homologous end joining, a critical cellular DNA–repair pathway. Biochemical experiments exploring alternate mechanisms show that group II intron reverse transcriptases can also template switch efficiently from one RNA template to a second RNA or DNA template, thereby directly linking the two template sequences. Our findings have implications for retotransposition and DNA repair mechanisms and potential biotechnological applications.

## Introduction

Mobile group II introns are site-specific retrotransposons that consist of a catalytically active intron RNA (ribozyme) and an intron-encoded protein (IEP), with reverse transcriptase (RT) activity [1]. Although they are found mainly in bacterial and organellar genomes, group II introns are thought to have played a major role in eukaryotic genome evolution as evolutionary ancestors of nuclear spliceosomal introns and retrotransposons in higher organisms [2]–[4]. Group II intron RNAs catalyze their own splicing via two sequential transesterification reactions that are the same as those for spliceosomal introns and yield an excised intron lariat with a branched 2′-5′ phosphodiester linkage [5], [6]. For mobile group II introns, the splicing reactions are assisted by the IEP, which binds specifically to the intron RNA and stabilizes the catalytically active RNA structure [7], [8]. The IEP then remains bound to the excised intron lariat RNA in a ribonucleoprotein particle (RNP) that promotes intron integration into new DNA sites [9], [10]. Intron integration is targeted to the ligated-exon junction in an intronless alleles in a process called “retrohoming”, but can also occur at lower frequency into ectopic sites that resemble the homing site in a process called “retrotransposition” or “ectopic retrohoming”. In both cases, the intron inserts into the new DNA site by a novel mechanism in which the excised intron lariat RNA fully reverse splices into a DNA strand and is reverse transcribed by the IEP, yielding an intron cDNA that is integrated into the genome by host enzymes [1], [10]–[14]. Retrohoming leads to the expansion of intron-containing alleles in a population, while ectopic retrohoming provides a means of intron dispersal to new sites.

Group II intron RNAs can also splice without branching by an alternate pathway, termed “hydrolytic splicing” [1]. In this pathway, the first transesterification, 5′-splice site cleavage, occurs by hydrolysis rather than branching, and the second transesterification yields ligated exons and an excised linear intron RNA. Hydrolytic splicing was first observed as a side reaction of group II intron self-splicing under non-physiological conditions [15], [16] and was demonstrated to occur *in vivo* by using a mutant yeast mitochondrial intron that was deleted for the branch-point A residue [17]. Some subclasses of group II introns lack the branch-point A residue and rely entirely on the hydrolytic mechanism for splicing *in vivo* [18], [19]. Linear group II intron RNAs can also be generated from excised intron lariat RNAs by debranching, which is believed to accelerate RNA turnover [20]. However, the physiological and evolutionary significance of hydrolytic splicing and linear group II intron RNAs have remained largely unclear.

**Figure 1:**
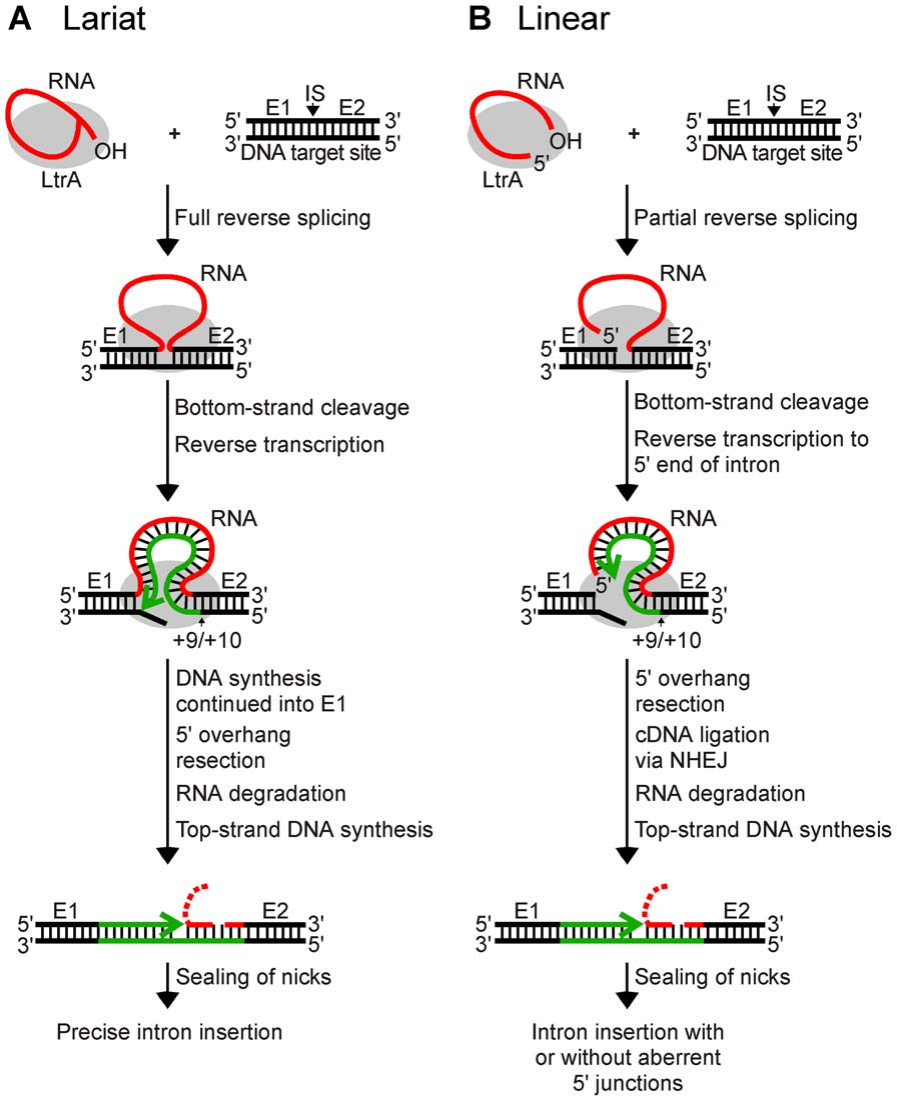
Models for retrohoming of Ll.LtrB group II intron lariat and linear RNAs. (A) Retrohoming of lariat RNA. RNPs containing lariat RNA recognize the DNA target site (ligated E1–E2 sequence) and carry out both steps of reverse splicing, resulting in insertion of the intron RNA between E1 and E2. The IEP uses its En domain to cleave the bottom strand between positions +9 and +10 of E2, and then uses the 3′ end of the cleaved DNA strand as a primer for reverse transcription of the inserted intron RNA. The resulting full-length intron cDNA is extended directly into E1 by continued DNA synthesis. Retrohoming is completed by a process that includes removal of the 5′ overhang on the bottom strand, degradation or displacement of the intron RNA template strand, top-strand DNA synthesis by a host DNA polymerase, and sealing of nicks by a host DNA ligase [21]. (B) Retrohoming of linear RNA. RNPs containing linear intron RNA recognize the DNA target site and carry out the first step of reverse splicing, resulting in ligation of the 3′ end of the intron RNA to the 5′ end of E2. The IEP then uses its En domain to cleave the bottom strand between positions +9 and +10, generating a primer for reverse transcription of the intron RNA, as in lariat RNA retrohoming. However, because the 5′ end of the linear intron RNA is unattached, the resulting cDNA cannot be extended directly into E1 and is instead linked to the 5′ exon DNA by an error-prone process that sometimes leads to precise insertion of the intron RNA, but often gives imprecise 5′ junctions due to deletion of E1 sequences, 5′-intron truncations, and/or insertion of extra nucleotide residues at the ligation junction. As for lariat RNA, retrohoming of the linear intron RNA is completed by degradation or displacement of the intron RNA template strand, top-strand DNA synthesis, and sealing of nicks by host enzymes. E1 and E2, 5′ and 3′ exon, respectively; CS, bottom-strand cleavage site; IS, intron-insertion site.

The *Lactococcus lactis* Ll.LtrB intron, which has been used extensively as a model system for studying group II intron mobility mechanisms, has a broad host range and is actively mobile in *Escherichia coli*, making it possible to use *E. coli* genetic approaches to dissect mobility pathways [11], [21]. The major retrohoming pathway used by the Ll.LtrB intron in *E. coli* is shown in Figure 1A. After promoting splicing, the Ll.LtrB IEP, denoted LtrA protein, remains bound to the excised intron lariat RNA in RNPs that recognize a DNA target site at the ligated-exon junction (denoted E1–E2) of an intronless allele. The intron lariat RNA initiates retrohoming by fully reverse splicing into the top DNA strand, leading to insertion of the intron RNA between the two DNA exons. The IEP then uses a DNA endonuclease activity to cleave the bottom strand and uses the 3′ DNA end at the cleavage site as a primer for reverse transcription of the inserted intron RNA. In *E. coli*, the resulting intron cDNA is integrated into the host genome by a mechanism that involves degradation of the intron RNA template strand by a host RNase H and second-strand DNA synthesis by a host DNA polymerase [11], [21]. In variations of this mechanism, Ll.LtrB and other group II introns can also retrohome without bottom-strand DNA cleavage by using a nascent strand at a DNA replication fork to prime reverse transcription of the intron RNA [14], [22], [23], and yeast mitochondrial group II introns retrohome by using recombination rather than DNA repair for cDNA integration [24].

Recently, while carrying out experiments to test whether microinjected group II intron RNPs could be used for gene targeting in *Xenopus laevis* oocyte nuclei and *Drosophila melanogaster* embryos, we found that linear as well as lariat group II intron RNAs can retrohome *in vivo* [25]. This finding was surprising because, unlike lariat RNAs, linear group II intron RNAs can carry out only the first reverse-splicing step, ligation of the 3′ end of the intron RNA to the 5′ end of the 3′- exon DNA [26], [27]. While reverse transcription of fully reverse-spliced intron RNA yields an intron cDNA that can be extended directly by continued DNA synthesis into the upstream exon (Figure 1A), reverse transcription of a partially reverse-spliced intron RNA yields an intron cDNA with an unattached 3′ end that must be linked to the upstream exon DNA in a separate step (Figure 1B). Sequencing of 5′-integration junctions showed that this step occurs by an error-prone process. Although some events result in the precise insertion of the intron between the two DNA exons, most give 5′-integration junctions with 5′-exon deletions, intron 5′-end truncations, insertion of extra nucleotides at the intron-exon junction, or indications of DNA repair via base pairing of microhomologies on opposite sides of the break [25], similar to ligation junctions resulting from double-strand break repair by non-homologous end joining (NHEJ) [28]–[31]. NHEJ activities have been found to contribute to the retrotransposition of LINE elements and other retrotransposons in eukaryotes [32], [33] and may be exploited preferentially by retrotransposons to gain advantage in genetic conflict with their hosts, which rely on these enzymes for survival [34]. Thus, although group II introns are alien to *Xenopus* and *Drosophila*, they could be utilizing mechanisms that contribute to the retrotransposition of resident retroelements in eukaryotes and could be subject to host defenses that evolved to counter or mitigate such retrotransposition.

Although NHEJ seemed the most likely mechanism for attachment of the free cDNA to the 5′ exon in linear intron RNA retrohoming, an alternate possibility was that the RT template switches to the 5′-exon DNA, either directly or following incorporation of extra nucleotide residues at the end of the cDNA, resulting in synthesis of a continuous DNA bottom strand containing intron and 5′-exon sequences. Both template switching and incorporation of extra nucleotide residues at the ends of cDNA have been found for other non-LTR-retroelement RTs [35]–[37]. Although we thought this possibility unlikely because group II intron RTs appeared to have low DNA-dependent DNA polymerase activity *in vitro* [21], template switching and non-templated nucleotide addition by group II intron RTs have not been investigated previously.

Here, we used *Drosophila melanogaster* mutants to investigate the contribution of NHEJ activities to linear intron RNA retrohoming and assessed the involvement of template switching by comparing junctions formed by this mechanism *in vitro* with those formed during linear intron RNA retrohoming *in vivo*. Our results indicate that linear intron RNA retrohoming occurs primarily by a novel variation of NHEJ that uses host enzymes, including DNA ligase 4 (Lig4) and DNA repair polymerase θ (PolQ), but is minimally dependent upon Ku.

## Results

### Retrohoming of linear and lariat RNA in *D. melanogaster* mutant embryos

**Figure 2:**
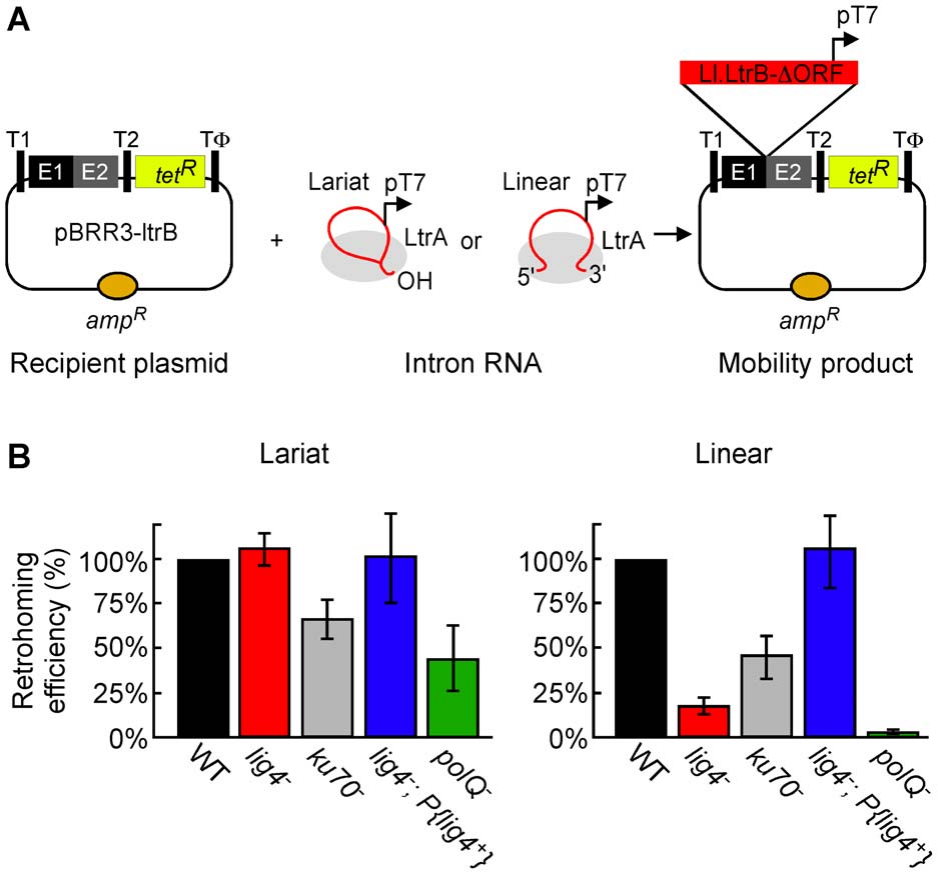
Retrohoming efficiencies of linear and lariat group II intron RNAs in wild-type and mutant *Drosophila*. (A) Microinjection assay for retrohoming of lariat and linear group II intron RNAs. *D. melanogaster* precellular blastoderm are microinjected with the Amp^R^-recipient plasmid pBRR3-ltrB, which contains an Ll.LtrB target site (ligated E1–E2 sequence) cloned upstream of a promoterless *tet* ^R^ gene, followed by separate microinjection of Ll.LtrB RNPs containing linear or lariat intron RNAs with a phage T7 promoter sequence inserted near their 3′ end. The embryos are incubated at 30°C for 1 h, during which the intron integrates into the target site in the recipient plasmid, placing the T7 promoter upstream of the promoterless *tet* ^R^ gene. Nucleic acids are then extracted and transformed into *E. coli* HMS174(DE3) for plating assays, and retrohoming efficiencies are calculated as the ratio of (Tet^R^+Amp^R^)/Amp^R^ colonies. T1 and T2, *E. coli rrnB* transcription terminators; Tφ, phage T7 transcription terminator. (B) Retrohoming efficiencies of lariat and linear Ll.LtrB RNPs in *D. melanogaster* wild-type (*w^1118^* and Or-R) and mutant embryos were determined, as described in panel A and Materials and Methods. The bar graphs show retrohoming efficiency in the indicated mutant embryos relative to that of wild-type embryos assayed in parallel in ten independent experiments with different combinations of strains (Table S1). The values are the mean for at least three independent determinations for each mutant, with the error bars indicating the standard error. The retrohoming efficiency of ≤0.5% wild type for linear RNPs in the *polQ* mutant is an upper limit, as only a single Tet^R^+Amp^R^ colony was recovered in three separate experiments, although 5′-intron integration junctions could be detected by using a more sensitive PCR assay in all experiments (see Figure 3).

To investigate the involvement of NHEJ factors, we compared lariat and linear group II intron retrohoming in *D. melanogaster* embryos with mutations in the genes encoding DNA ligase 4 (Lig4), Ku70, and the DNA repair polymerase θ (PolQ) [38], [39]. For these experiments, we used a plasmid-based retrohoming assay in which an Ll.LtrB-ΔORF intron with a phage T7 promoter sequence inserted near its 3′ end integrates into a target site cloned in an Amp^R^-recipient plasmid upstream of a promoterless *tet* ^R^ reporter gene, thereby activating that gene (Figure 2A) [25], [26], [40]. The recipient plasmid was injected into the posterior of precellular blastoderm stage embryos, followed within 5 min by injection of lariat or linear RNPs, which were reconstituted *in vitro* from the purified IEP and intron RNA (see Materials and Methods). After incubating the embryos for 1 h at 30°C, nucleic acids were extracted and transformed into an *E. coli* strain (HMS174(DE3)), which expresses T7 RNA polymerase. The transformed bacteria were then plated on medium containing ampicillin or ampicillin and tetracycline, and mobility efficiencies were quantified as the ratio of (Tet^R^+Amp^R^)/Amp^R^ colonies.

Figure 2B compares the retrohoming efficiencies of lariat and linear intron RNAs in wild-type and mutant embryos, based on parallel assays in ten separate experiments (summarized in Table S1). For each strain in each experiment, 80 injected embryos were pooled prior to extracting nucleic acids and transforming them into *E. coli*. The results for the *lig4* mutant show the retrohoming efficiency of the lariat intron was unchanged, whereas the retrohoming efficiency of the linear intron was decreased strongly (18% wild type), but could be restored to wild-type levels by ectopic expression of Lig4 from an integrated P-element (*lig4^−^; P{lig4^+^}* embryos). In the *polQ* mutant, the retrohoming efficiency of the linear intron was decreased to ≤0.5% of wild type, compared to 27% wild type for lariat RNPs. Finally, the *ku70* mutant, a *trans*-heterozygote of two putative null alleles (see Materials and Methods), showed only moderately decreased retrohoming efficiencies for both lariat and linear intron retrohoming (67% and 46% wild type, respectively). The latter result was surprising because Ku and Lig4 ordinarily function together in the same NHEJ pathway [41], [42].

The strong differential inhibition of linear compared to lariat RNA retrohoming in the *lig4* and *polQ* mutants supports models in which these enzymes function directly at unique steps in this process, presumably by providing the DNA ligase and repair DNA polymerase activities needed to link the intron cDNA to the upstream exon. The similar moderate decreases in lariat and linear intron retrohoming efficiency in the *ku70* mutant could reflect that Ku functions at a common step in both pathways or could be an indirect effect (see Discussion).

### Comparison of 5′-integration junctions from linear intron RNA retrohoming in wild-type and *lig4* and *ku70* mutant embryos

*D. melanogaster* uses at least two NHEJ pathways to repair double-strand breaks: classical NHEJ (C-NHEJ), which is dependent upon Lig4 and Ku70, and alternate end-joining (alt-EJ), which operates without either factor and could be a mixture of different pathways [31], [38], [39], [43]. In a genetic assay for repair of double-strand breaks induced in the germline by the meganuclease I-SceI, *lig4* and *ku70* mutants inhibited NHEJ activity by 76–78%, leaving 22–24% residual activity that was attributed to alt-EJ [43]. Our finding above that the *lig4* mutant shows similar degrees of inhibition and residual activity for linear intron retrohoming (82% and 18%, respectively), most simply suggests that Lig4-independent retrohoming occurs by using the alt-EJ pathway or components thereof.

**Figure 3:**
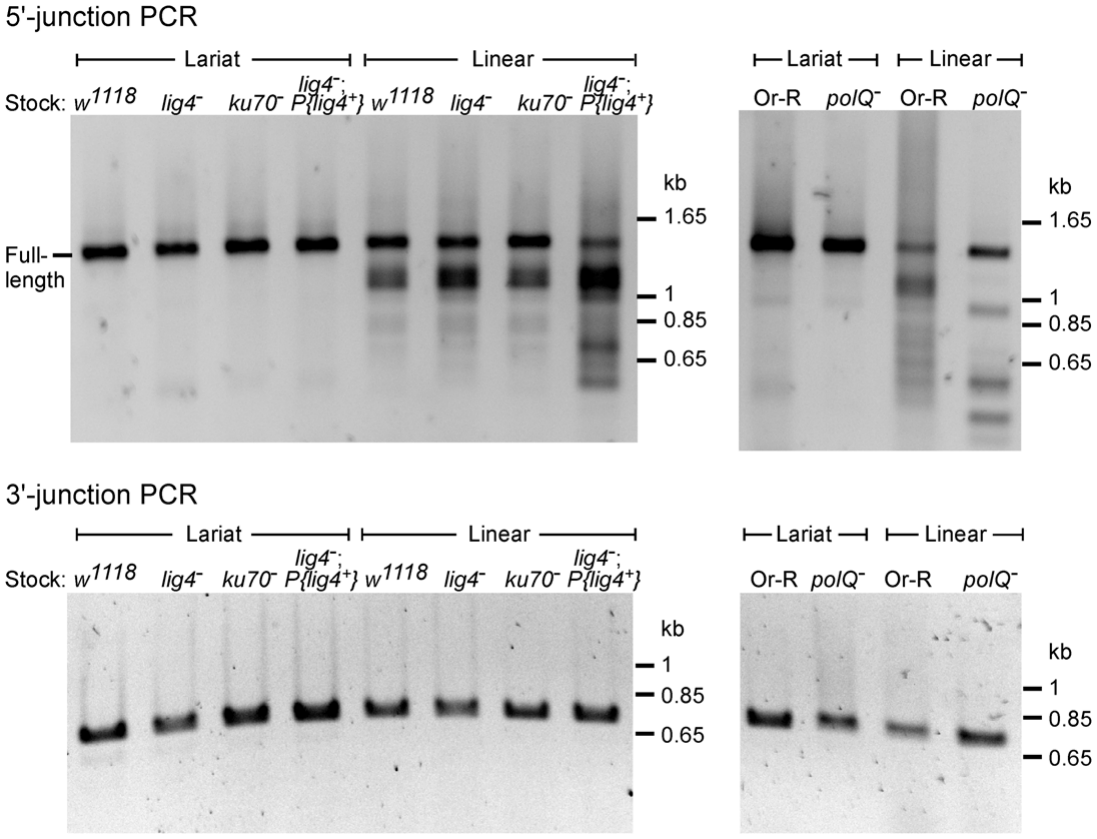
PCR analysis of integration junctions from lariat and linear intron RNA retrohoming in wild-type and mutant strains. Retrohoming assays with lariat and linear RNPs were done as described in Figure 2A and Materials and Methods, and DNA was extracted from 80 pooled embryos for each strain. 5′- and 3′-integration junctions were amplified by PCR, using primers that flank the junction (5′junction, forward primer P1 and reverse primer LtrB933a; 3′ junction, forward primer P3 and reverse primer P4; see Materials and Methods). The PCR products were analyzed in a 1% agarose gel, which was stained with ethidium bromide. Precise 5′ and 3′ junctions for lariat intron RNA retrohoming and precise 3′ junctions for linear intron RNA retrohoming were confirmed by sequencing junctions from at least 10 randomly selected Tet^R^+Amp^R^ colonies or PCR products from pooled embryos for all strains (not shown).

**Figure 4:**
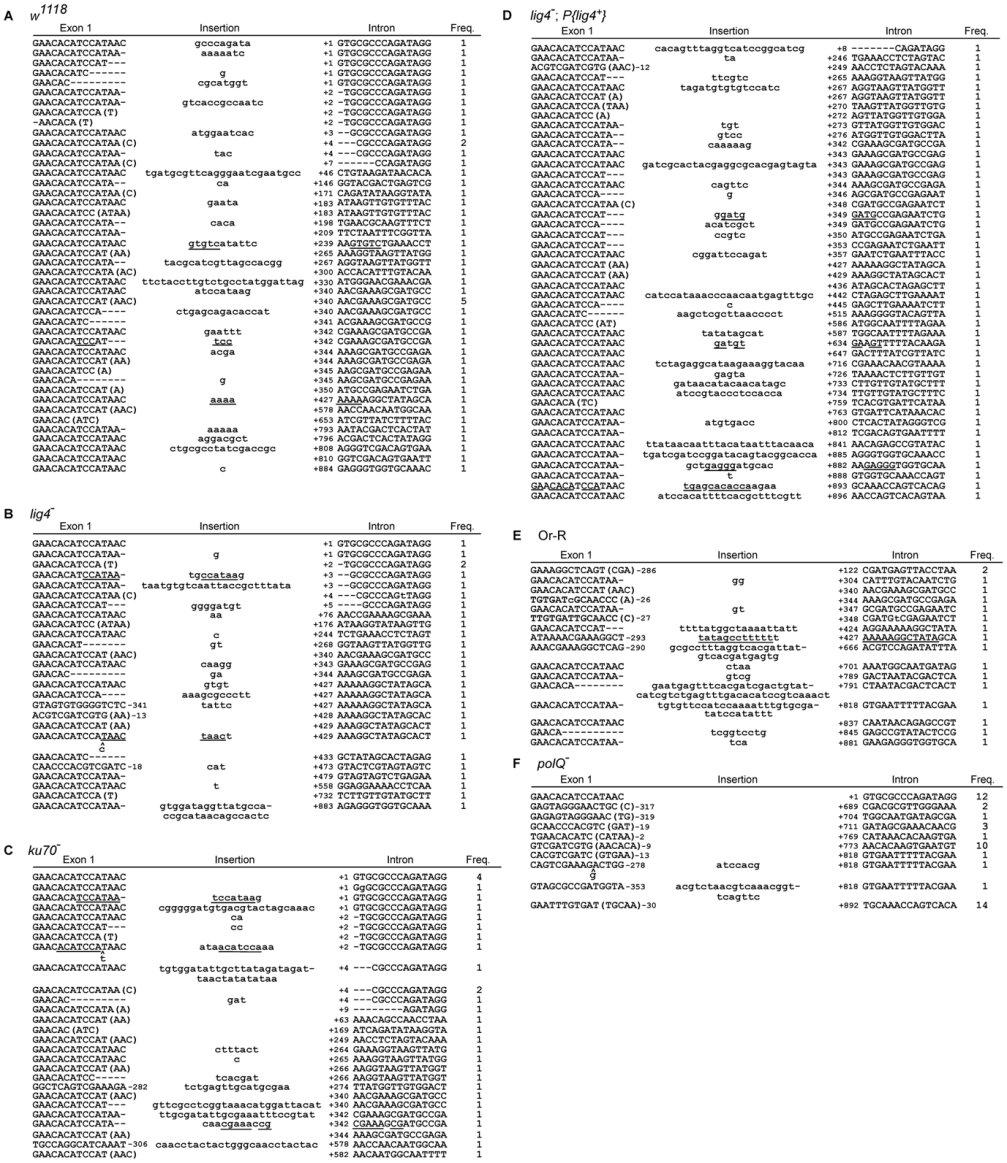
Sequences of 5′-integration junctions from linear intron RNA retrohoming in wild-type and mutant strains. 5′-integration junctions of DNA extracted from 80 pooled embryos for each strain were amplified by PCR, as described in Figure 3, then TOPA-TA cloned, amplified by colony PCR, and sequenced, as described in Materials and Methods. (A) wild-type *w^1118^*; (B) *lig4* ^−^; (C) *ku70* ^−^; (D) *lig4^−^; P{lig4^+^};* (E) wild-type Or-R; (F) *polQ* ^−^. Inserted or mutant nucleotide residues are shown in lower case letters; microhomologies between intron and exon end sequences prior to ligation are shown in parentheses; and inserted sequences that match or are complementary to nearby 5′-exon or intron sequences are underlined. Freq., frequency of occurrence.

Previous studies showed that the DNA repair junctions resulting from alt-EJ in *Drosophila ku70* and *lig4* mutants are generally similar to those for C-NHEJ [31], although in some assays, the *lig4* mutant gave somewhat increased frequencies of junctions with extra nucleotide additions (55–63% compared to 30–36% for wild type [38], [39]). To further investigate whether Lig4-independent retrohoming occurs via alt-EJ, we compared 5′- and 3′-intron integration junctions resulting from linear intron retrohoming in the mutant and two commonly used wild-type strains (*w^1118^* and Or-R) by PCR using primers flanking the junctions, followed by cloning and sequencing of the PCR products (Figure 3 and Figure 4).

The 5′- and 3′-integration junctions from lariat intron retrohoming and the 3′-integration junctions from linear intron retrohoming result from reverse-splicing reactions (see Figure 1), and as expected, the PCRs for these junctions gave single prominent products, with no differences between the wild-type and mutant strains (Figure 3, bottom gels; in each case, the expected precise junction sequence was confirmed by sequencing; see legend for details). By contrast, the 5′-integration junctions resulting from linear intron RNA retrohoming were heterogeneous in all strains, with a major band of the size expected for full-length intron insertion and smaller bands, which appeared most prominent in the *lig4^−^; P{lig4^+^}* and *polQ* ^−^ embryos (Figure 3, top gels).

**Figure 5:**
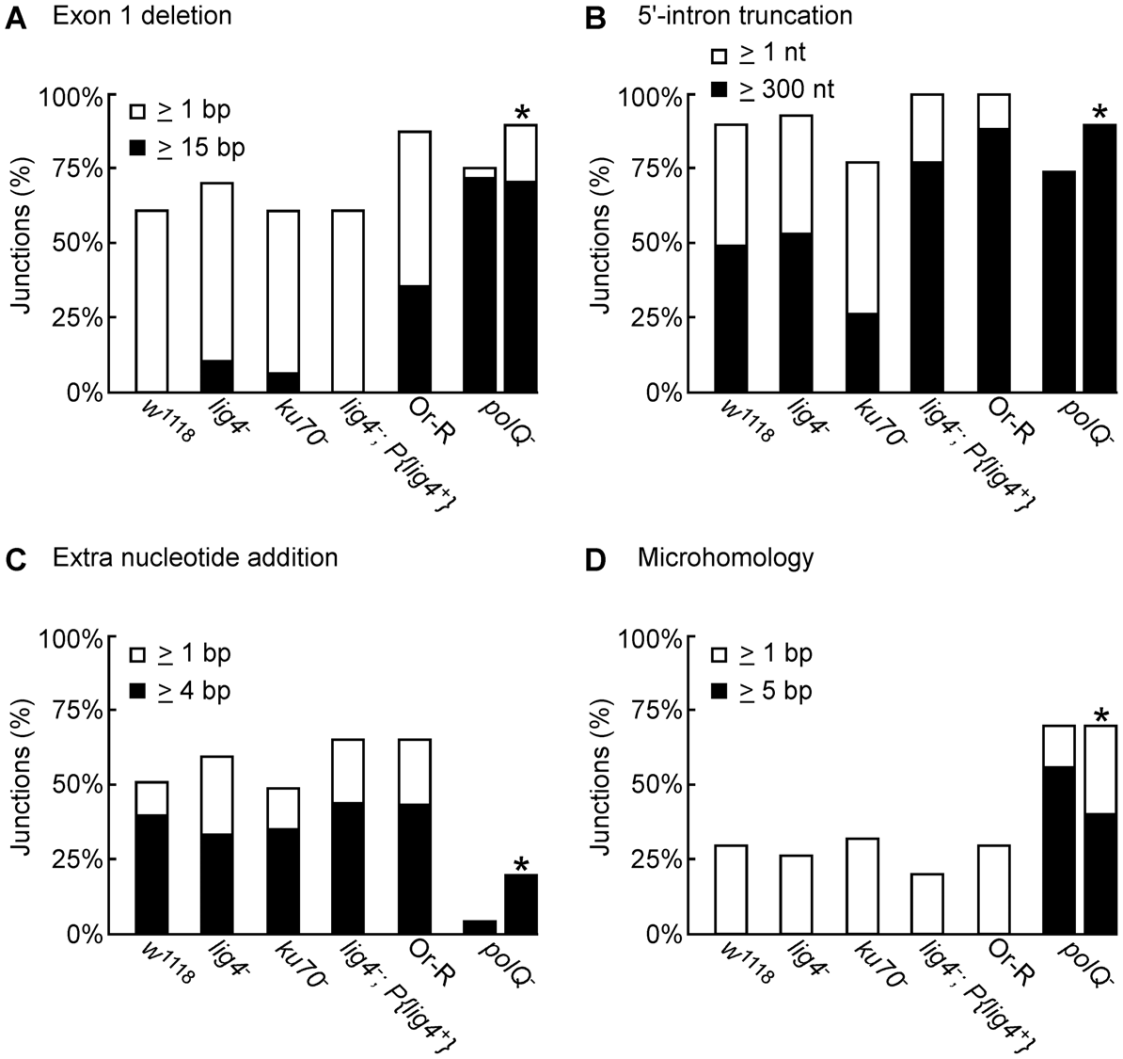
Characteristics of 5′-integration junctions resulting from linear intron RNA retrohoming in wild-type and mutant strains. The bar graphs show the percentage of 5′-integration junctions with (A) exon 1 deletions, (B) 5′-intron truncations, (C) extra nucleotide additions, and (D) microhomologies in the indicated strains. For the *polQ* ^−^ embryos, where the number of unique junction sequences recovered was smaller than for the other strains, the percentage of junctions having the indicated characteristics was calculated both as a percentage of total junctions (left bar) and a percentage of unique junctions (right bar, asterisk).

DNA sequences of the 5′-integration junctions resulting from linear intron retrohoming in wild-type *w^1118^* and *lig4^−^*, *ku70^−^*, and *lig4^−^; P{lig4^+^}* embryos are summarized in Figure 4A–4D, and their characteristics are compared by the bar graphs in Figure 5. As found previously [25], the 5′-integration junctions for linear intron RNA retrohoming in the wild-type embryos were heterogeneous with different combinations of 5′-exon deletions, 5′-intron truncations, and extra nucleotide additions (Figure 4A). Some of the junctions show evidence of DNA repair at regions of microhomology (parentheses), and in some cases, the extra nucleotides inserted at the junctions match and were presumably copied from neighboring sequences in the 5′ exon or intron (underlined).

The 5′ junctions resulting from linear intron retrohoming in the *lig4* ^−^, *ku70* ^−^, and *lig4^−^; P{lig4^+^}* mutants were generally similar to those in the wild type *w^1118^*, the more closely related wild-type strain, with no large differences in the percentage of junctions with 5′-exon deletions, 5′-intron truncations, extra nucleotide additions, or microhomologies (Figure 4 and Figure 5). Compared to the other strains assayed in parallel, the proportion of long 5′-intron truncations appears to be somewhat lower in the *ku70^−^* embryos and higher in the *lig4* ^−^ and *lig4^−^; P{lig4^+^}* embryos, but the significance of these findings is unclear, as the differences were not large and the proportions of full-length and shorter 5′-junction products in each stock were somewhat variable in different experiments. The similarity of the junction sequences resulting from linear intron RNA retrohoming in the *lig4* and *ku70* mutants to those resulting from double-strand break repair in these mutants [31], [39] supports the hypothesis that Lig4-independent linear intron retrohoming occurs predominantly by using components of the alt-EJ pathway.

### The *polQ* mutation decreases extra nucleotide addition and increases long microhomologies at 5′-integration junctions

The DNA repair polymerase θ (PolQ) has been shown to function in DNA end-joining repair in *Drosophila*, including a role for extra nucleotide addition at the repaired junctions [38]. To investigate the function of PolQ in linear intron RNA retrohoming, we compared the sequences of 5′-intregration junctions from parallel assays of this process in wild-type Or-R and *polQ^−^* embryos (Figure 4E and 4F, Figure 5). Because the number of unique junction sequences recovered from the *polQ* mutant was lower than those for the other strains and some of these junctions were represented multiple times, we calculated the proportion of junctions with different characteristics in Figure 5 relative to both the total number of junctions (left bars) and the total number of unique junctions sequences (right bars, asterisks). Both comparisons show that that the *polQ* mutation decreases the proportion of junctions containing extra nucleotide residues (4% of total junctions and 20% of unique junctions compared to >64% of junctions in wild-type Or-R and >48% in all other strains analyzed), as expected from the known function of PolQ. Further, the 5′ junctions from the *polQ* ^−^ embryos have a higher frequency of long (≥15 bp) 5′-exon deletions (72% of total junctions and 80% of unique junctions compared to 29% in wild-type Or-R) and a dramatically increased frequency of long (≥5 nt) microhomologies between exon and intron sequences (56% of total junctions and 40% of unique junctions compared to none among 170 total junctions from all the other strains analyzed). This increased frequency of long microhomologies may reflect that they are more stringently required for annealing of the 3′ end of the cDNA to the upstream exon in the absence of PolQ. We note that among the unique junction sequences from the *polQ* mutant, two with large deletions were recovered ≥10 times each. Although we cannot exclude that the repeated recovery of these junctions reflects differential amplification by PCR, both have ≥5 nt microhomologies that could have been used preferentially for annealing in multiple events, and indeed one of these junctions (Figure 4F bottom sequence) comprised 6 of 12 recovered junctions in an additional, separate experiment (data not included in Figure 4F). Considered together, the junction sequences indicate that PolQ functions in extra nucleotide addition to the 3′ end of the cDNA during linear intron RNA retrohoming and that this extra nucleotide addition may be critical for generating microhomologies that enable annealing between the 3′ end of the cDNA and the upstream exon DNA. Further, the strongly decreased frequency of linear intron RNA retrohoming in the *polQ* mutant indicates that PolQ functions in both the Lig4-dependent and Lig4-independent retrohoming pathways.

### The Ll.LtrB RT can template switch from the 5′ end of linear intron RNA to the upstream exon

Although the residual linear intron RNA retrohoming events in the *lig4* mutant can be accounted for by Lig4-independent (alt-EJ) NHEJ, it remained possible that template switching of the RT from the 5′ end of the intron RNA directly to the 3′ end of the 5′-exon DNA contributes to this process. Previous studies have shown that other non-LTR retroelement RTs are proficient at template switching directly to the 3′ end of a template strand with little or no complementarity to the cDNA end and that these events can be accompanied by extra nucleotide addition at the junctions, as found for NHEJ [35], [36], [37], [44].

**Figure 6:**
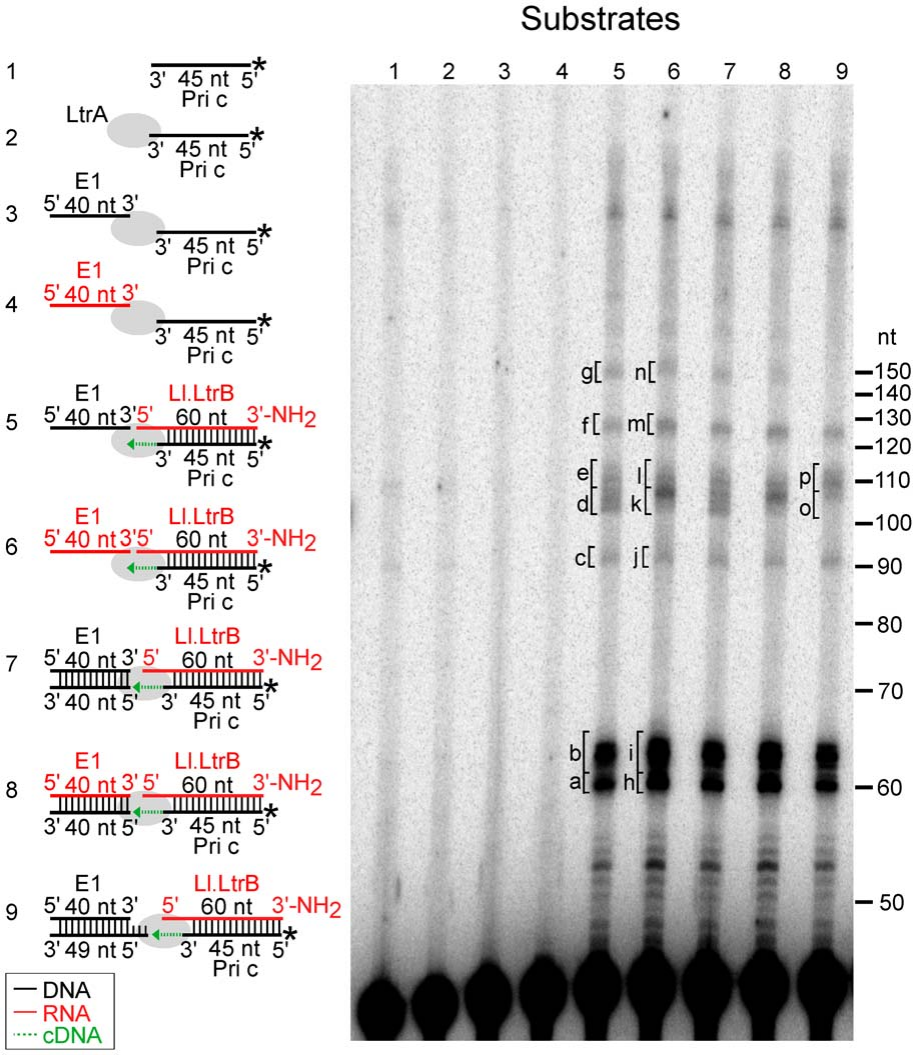
Template switching of LtrA from the 5′ end of the Ll.LtrB intron RNA to exon 1 DNA or RNA. The Ll.LtrB intron RT (LtrA protein; 40 nM) was incubated with artificial substrates corresponding to the 5′ end of Ll.LtrB intron (Ll.LtrB RNA; 40 nM) with an annealed 5′-^32^P-labeled DNA primer c (Pri c; 44 nM) in presence of exon 1 (E1) DNA or RNA (40 nM; black and red, respectively), as diagrammed in schematics to the left of the gel. The substrates were incubated with dNTPs (200 µM) in reaction medium containing 450 mM NaCl, 5 mM MgCl_2_, 20 mM Tris-HCl, pH 7.5, and 1 mM DTT for 30 min at 30°C. After terminating the reaction by extraction with phenol-CIA, the products were analyzed in a denaturing 15% polyacrylamide gel. Lanes (1) and (2) ^32^P-labeled Pri c incubated without and with LtrA, respectively; (3) and (4) LtrA incubated with ^32^P-labeled Pri c and E1 DNA or RNA, respectively; (5) and (6) LtrA incubated with Ll.LtrB RNA with annealed ^32^P-labeled Pri c and E1 DNA or RNA, respectively; (7–9) LtrA incubated with Ll.LtrB RNA with annealed ^32^P-labeled Pri c and E1 DNA or RNA with annealed complementary DNA oligonucleotides to leave a blunt end (exon 1 AS) or a 5′-bottom-strand overhang (exon 1 AS+9). Bands excised for sequencing (Figure 7) are indicated in the gel. In the schematics, DNA and RNA oligonucleotides are shown in black and red, respectively; LtrA is shown as a gray oval; and the direction of cDNA synthesis is indicated by a green arrow. The numbers to the right of the gel indicate the positions of 5′-end labeled size markers (10-bp DNA ladder, Invitrogen).

To determine if a template-switching mechanism could be responsible for the manner of 5′ junctions observed during linear intron retrohoming, we carried out biochemical assays using small artificial substrates that simulate the situation at the 5′-integration junction just prior to completion of intron cDNA synthesis (Figure 6). The primary substrate consists of a 60-nt RNA template (Ll.LtrB RNA), whose 5′ end corresponds to that of the Ll.LtrB intron, with a 45-nt DNA primer representing the nascent cDNA (primer c) annealed to its 3′ end. The Ll.LtrB RT (LtrA) initiates reverse transcription of the intron RNA template from the annealed DNA primer and extends it to the 5′ end of the Ll.LtrB RNA template, where it can then switch to a second 40-nt DNA or RNA template with the nucleotide sequence of exon 1 (E1 RNA or DNA, red and black, lanes 5 and 6, respectively). The 3′ end of the Ll.LtrB RNA has an aminoblock to impede the RT from switching to a second molecule of the initial template.

Figure 6, lanes 5 and 6 show that the Ll.LtrB RT efficiently extends the annealed primer c (Pri c) to the end of the intron RNA template, yielding major labeled products of ∼60-nt, which were resolved as a doublet, along with smaller amounts of larger products of the size expected for template switching to the exon 1 (E1) DNA or RNA (∼100 nt) or to a second molecule of Ll.LtrB RNA despite the presence of the aminoblock (∼120 nt). Controls show that no labeled products were detected after incubating the RT with primer c in the presence or absence of the exon 1 RNA or DNA (lanes 2–4).

**Figure 7:**
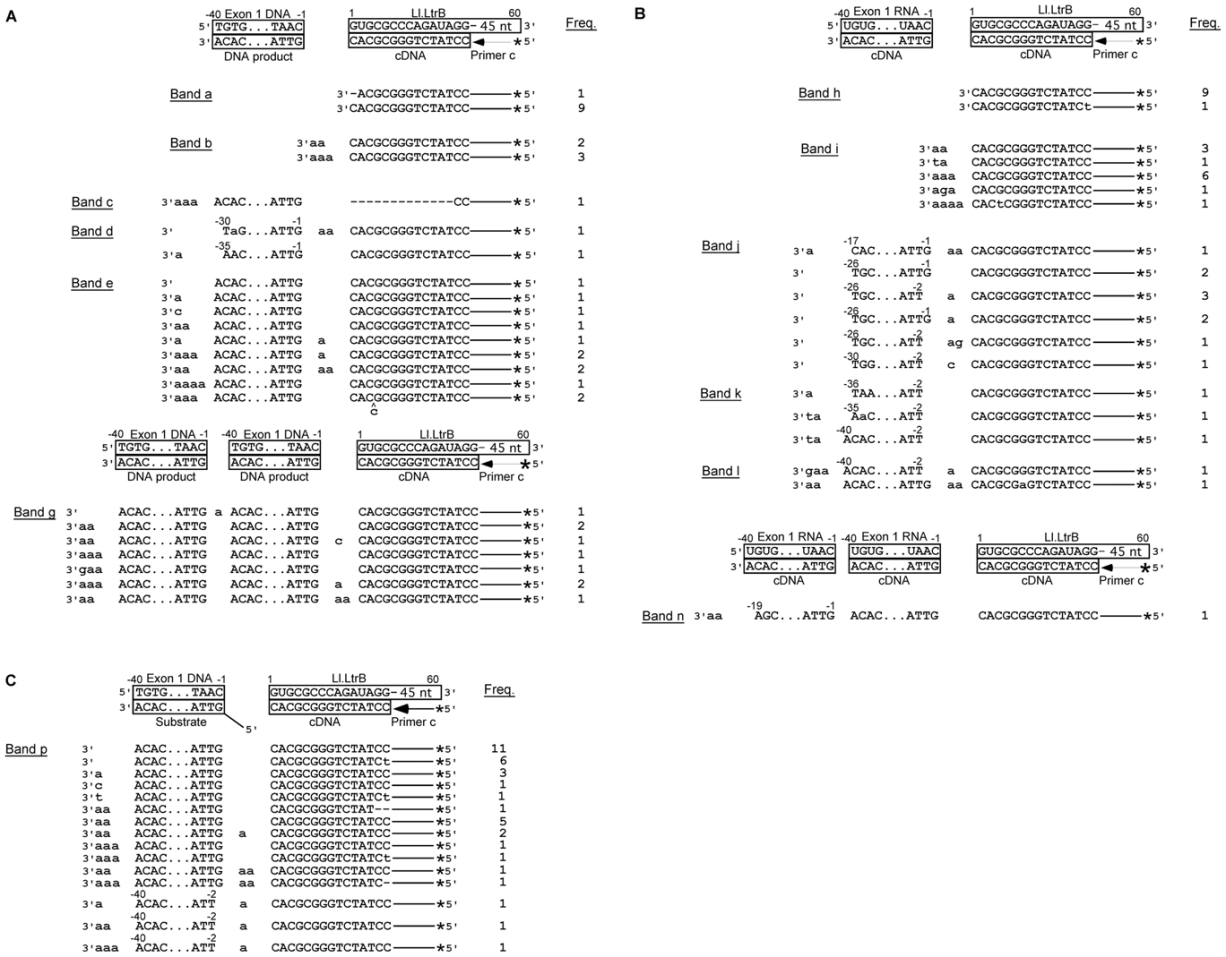
DNA sequences resulting from template switching of LtrA from Ll.LtrB RNA to exon 1 DNA or RNA. (A) and (B) Sequences of DNA products resulting from template switching of the Ll.LtrB RT (LtrA protein) from the 5′ end of the Ll.LtrB intron RNA template/Pri c DNA substrate to exon 1 DNA (lane 5) or RNA (lane 6), respectively. (C) Sequences of DNA products resulting from template switching of the Ll.LtrB RT from the 5′ end of Ll.LtrB RNA/primer c substrate to double-stranded exon 1 DNA with a 5′ bottom-strand overhang (AS+9; lane 9). Bands were excised from the gel, cloned, and sequenced, as described in Materials and Methods. The substrate and expected cDNA or DNA product sequences are shown boxed above each set of experimentally determined DNA product sequences. Extra or mutant nucleotide residues are shown in lower case letters. Freq., frequency of occurrence; *, ^32^P-label at 5′ end of primer c.

Cloning and sequencing of the gel bands confirmed that the major ∼60-nt products (bands a and b in lane 5 and h and i in lanes 6) correspond to cDNAs extending to or near the 5′ end of the intron RNA, with the doublet reflecting the addition of extra nucleotide residues, mostly A-residues, to the 3′ end of the cDNA upon reaching the end of the Ll.LtrB RNA template (Figure 7A and 7B). Such non-templated nucleotide addition is a common property of DNA polymerases and RTs [35], [36], [45]–[48].

The first set of larger gel bands (90–110 nts; band c–e in lane 5 and j–l in lane 6) corresponds to products resulting from template switching from the 5′ end of the intron to the 3′ end of exon 1 DNA or RNA (Figure 7A and 7B), as well as products resulting from template switching to the 3′ end or internal regions of the Ll.LtrB intron (Figure S1). Many of the template switches to exon 1 DNA occurred seamlessly, but small numbers of extra nucleotide residues, mostly A residues, were found at some junctions, as well as at the 3′ end of the cDNAs (Figure 7A; bands c–e). The template switches to exon 1 RNA showed similar characteristics, but with a higher proportion of junctions containing extra nucleotide residues (61% compared to 33% for exon 1 DNA; Figure 7B; bands j–l).

The second set of larger bands (120–140 nts; bands f and g in lane 5 and m and n in lane 6) contains products resulting from two sequential template switches to exon 1 DNA or RNA (Figure 7A and 7B, respectively) and/or the Ll.LtrB RNA (Figure S1). These products of multiple template switches have characteristics similar to those resulting from a single template switch, including addition of extra nucleotide residues, mostly A residues, at some template-switching junctions and at the 3′ ends of the cDNAs.

The above results were obtained under reaction optimized for reverse transcription by the Ll.LtrB RT *in vitro* (450 mM NaCl, 5 mM Mg^2+^), the high salt concentration helping to stabilize free protein and minimize aggregation of this RT [9]. However, similar results were obtained for template-switching reactions under near-physiological salt conditions (100 or 200 mM KCl, 5 mM Mg^2+^). Although the RT activity of the protein was lower under these conditions, the gel profiles show roughly equal levels of template switching to exon 1 RNA and DNA (Figure S2), and sequencing of the products showed similar template-switching junctions and patterns of non-templated nucleotide addition (Figure S3).

Finally, we tested whether the Ll.LtrB RT could template switch to double-stranded exon 1 DNA or RNA with an annealed bottom-strand DNA leaving either a blunt end or a 5′ bottom-strand overhang identical to that generated *in vivo* by the staggered double-strand break accompanying group II intron insertion (Figure 1; complete annealing confirmed by native gel analysis; Figure S4). Neither of these configurations significantly decreased the formation of the 100-nt product resulting from template switching to exon 1 DNA or RNA (Figure 6, lanes 7–9). DNA sequencing confirmed the template switch to double-stranded E1 DNA with a 5′-bottom-strand overhang and showed that this template switch was seamless in most cases (Figure 7C). The sequencing also showed several instances in which the template switch occurred to the penultimate rather than the 3′ terminal residue of exon 1 (Figure 7C), as well as template switches to Ll.LtrB RNA and the bottom-strand overhang oligonucleotide (Figure S5). Template switching to the penultimate nucleotide residue was not seen for single-strand acceptor DNA templates and could be related to the presence of the complementary DNA strand.

Together, the biochemical assays show that the Ll.LtrB RT can template switch from the 5′ end of the intron RNA to exon 1 and surprisingly that template switching is similarly efficient regardless of whether the exon 1 template is RNA or DNA or single- or double-stranded. However, the junctions resulting from template switching differ from those generated during retrohoming of linear intron RNA *in vivo* in that extra nucleotide additions are uniformly short and mostly A-residues.

## Discussion

**Figure 8:**
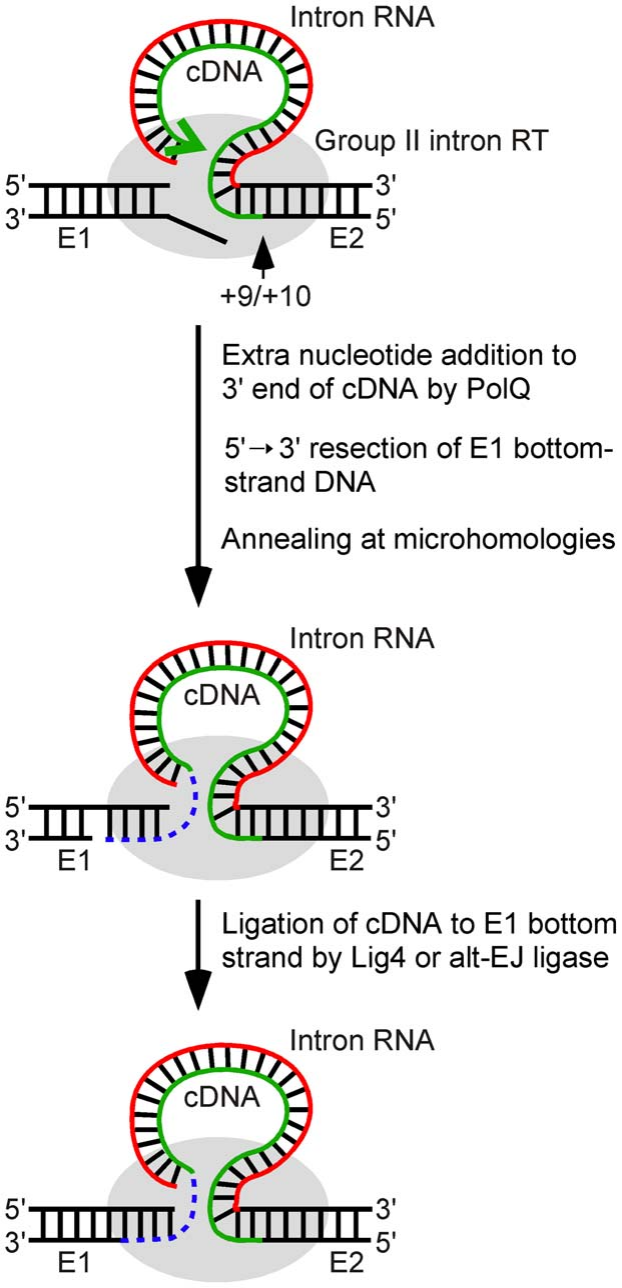
Model for ligation of the intron cDNA to exon 1 during linear group II intron retrohoming. Partial reverse splicing of the linear intron RNA into the DNA target site is followed by bottom-strand cleavage between E2 positions +9 and +10 and synthesis of a cDNA of the attached linear intron RNA. After cDNA synthesis reaches the 5′ end of the intron RNA template, extra nucleotide residues are added to the 3′ end of the cDNA generating microhomologies that enable annealing of the cDNA strand to the top strand of E1. In *Drosophila*, the repair DNA polymerase θ is the major enzyme responsible for this extra nucleotide addition, but some extra nucleotide addition may also be done by other host DNA polymerases or by the Ll.LtrB RT. The annealing of the cDNA requires unwinding and/or resection of the bottom strand, leading to loss of the bottom-strand 5′ overhang resulting from the initial double-strand break by the group II intron RNP. In the final step, the annealed cDNA is ligated to the bottom strand of E1 by DNA ligase 4 or an alternate ligase. The retrohoming of the linear intron RNA is completed by degradation or displacement of the intron RNA template strand, second-strand DNA synthesis, and sealing of nicks by host enzymes. E1 and E2, 5′ and 3′ exons, respectively.

Considered together, our results lead to the model shown in Figure 8 for the key steps in linear intron RNA retrohoming. The finding of strong differential inhibition of linear relative to lariat intron retrohoming in *D. melanogaster* mutants indicates that the NHEJ factor Lig4 is the predominant enzyme involved in ligating the intron cDNA to the upstream exon and that extra nucleotide addition by the DNA repair polymerase θ (PolQ) also plays a crucial role. Although Lig4 and PolQ appear to be the major enzymes playing these roles in *D. melanogaster*, residual linear intron RNA retrohoming with extra nucleotide addition occurs in both the *lig4* and *polQ* mutants, indicating that other DNA ligases and polymerases can serve as backups that perform the same functions at lower efficiency. Biochemical experiments show that another possible Lig4-independent mechanism, template switching by the group II intron RT from the 5′ end of the intron RNA directly to the upstream exon DNA, is possible but gives junctions differing from the majority of those *in vivo*. It seems likely that the mechanism elucidated here involving host DNA ligases and repair polymerases is also used for linear intron RNA retrohoming in *Xenopus laevis*, where we observed similar 5′-integration junctions [25], and more generally, in other eukaryotes, including mammalian cells, where it could have implications for group II intron-based gene targeting. This mechanism also provides a possible means for proliferation of non-branching group II introns in prokaryotes, some of which encode a Ku homolog and ATP-dependent DNA ligases along with DNA repair polymerases and use them in NHEJ pathways related to those of higher organisms [49]. Additionally, features of this mechanism, including the use of both Lig4-dependent and alt-EJ and the requirement for extra nucleotide addition to the cDNA end by a DNA repair polymerase, may be used to promote retrotransposition and mitigate DNA damage caused by LINE elements and other retrotransposons [33], [50]–[52].

The involvement of Lig4 in linear intron RNA retrohoming in *Drosophila* is indicated by the findings that a *lig4* mutation decreases the retrohoming efficiency of linear intron RNA by ∼80% while having no effect on the retrohoming of lariat RNA, and that the decreased retrohoming efficiency of the linear intron in the mutant could be restored to wild-type levels by ectopic expression of Lig4 from a P-element insertion. Most if not all of the residual linear intron RNA retrohoming in the *lig4* mutant appears to occur by using components of the alt-EJ pathway, as judged both by similar levels of activity and characteristics of the cDNA ligation junction, particularly patterns of extra nucleotide and the use of microhomologies (see Results). In *Drosophila*, C-NHEJ and alt-EJ give generally similar double-strand break repair junctions, albeit with quantitative differences in the frequency of extra nucleotide addition in some assays [31], [38], [39], whereas in yeast or mammalian cells, alt-EJ junctions show increased deletion lengths and use of microhomologies [53]–[55].

The involvement of PolQ in linear intron RNA retrohoming is indicated by the findings that a PolQ mutation decreases the retrohoming efficiency by >99% and substantially decreases the frequency of 5′-integration junctions having extra nucleotide residues (4–20% of junctions compared to 67% for wild-type Or-R assayed in parallel and >48% in all other strains; Figure 4 and Figure 5). The mutation also increases the frequency of junctions with long (≥15 bp) 5′-exon deletions and long (≥5 nt) microhomologies. The latter increase is particularly striking, as such long microhomologies were found at 56% of the total and 40% of the unique junction sequences from the *polQ* mutant, but were not found at junctions (170 total) from any of the other strains analyzed (Figure 4 and Figure 5). The residual extra nucleotide addition in the *polQ* mutant, which was also seen at 15–20% of junctions in end-joining assays [38], [39], could be due to small amounts of the enzyme remaining in the mutant, which has an unidentified expression defect, or to an alternate DNA polymerase. The very strong decrease in linear intron RNA retrohoming efficiency in the *polQ* mutant (>99%) indicates that PolQ functions in both the Lig4-dependent and Lig4-independent retrohoming pathways.

PolQ could potentially play at least two roles in linear intron RNA retrohoming. First, extra nucleotide addition to the 3′ end of the cDNA by PolQ may be critical for generating microhomologies that can base pair with the upstream exon to facilitate DNA ligation. Second, PolQ contains a putative DNA helicase domain that could also contribute to retrohoming by promoting base pairing between microhomologies at the cDNA end and the upstream exon, either by annealing the cDNA end to complementary exon sequences or by unwinding the exon DNA strands, making the top strand more accessible to base pairing [38]. The increased frequency of long 5′-exon deletions in the *polQ* mutant may reflect a delay in cDNA attachment due to lack of suitable microhomologies and/or impaired annealing of complementary cDNA ends to the top strand. The striking increase in the frequency of long microhomologies at the 5′ junctions in the *polQ* mutant (see above) indicates that an alternate annealing mechanism exists in the *polQ* mutants, but that it is more dependent upon longer microhomologies between exon and intron sequences than the PolQ-assisted mechanism.

The function, if any, of Ku in linear intron RNA retrohoming is unclear. The finding that *ku70* mutations moderately inhibit retrohoming of both linear and lariat intron RNA (54 and 33% inhibition, respectively) could reflect either that Ku contributes to both pathways or that Ku mutations affect one or both pathways indirectly. The Ku protein interacts with a stem-loop region of the RNA component of yeast and human telomerase [56]–[58], and it is possible that Ku may similarly bind to linear or lariat group II intron RNAs to protect them from degradation and/or recruit other DNA repair enzymes to the site. An alternate possibility is that Ku affects retrohoming efficiency indirectly by contributing to the repair of double-strand breaks induced by the intron RNP in the recipient plasmids. In yeast mitochondria, double-strand breaks resulting from abortive retrohoming events are substantially more frequent than completed integrations [59]. If not repaired correctly, such double-strand breaks could lead to loss of functional recipient plasmid target sites, which would appear as decreased retrohoming efficiencies in our assay. A similar indirect effect, involving repair of double-strand breaks in the recipient plasmid could also account for the moderate inhibitory effect of the *polQ* mutation on lariat intron RNA retrohoming.

Lig4 is ordinarily recruited to DNA breaks by Ku, and *D. melanogaster* mutations in either *lig4* or *ku70* give similar decreases in NHEJ efficiency, suggesting that Lig4 acts exclusively in Ku-dependent NHEJ [41]–[43]. By contrast, we find that linear intron RNA retrohoming is more strongly inhibited by a *lig4* mutation than by putative null mutations in *ku70* (82 and 54% inhibition, respectively). Even assuming that the inhibition by the *ku70* mutations is a direct effect, these findings most simply suggest that a substantial proportion of linear intron RNA retrohoming events are promoted by Lig4 in the absence of Ku.

Unlike a conventional double-strand break, the double-strand break formed during linear intron RNA retrohoming has an RNA attached to one of the DNA ends, and this difference could potentially affect the recruitment and use of NHEJ activities. We noted previously that group II intron RNPs bind to both the 5′- and 3′-exons during retrohoming, and such bridging of the ligation junction could impede access and decrease the need for Ku to cap the broken DNA ends [25]. Additionally, the attached intron RNA could contribute directly to the recruitment of NHEJ activities. The interaction of Ku with telomerase RNA noted above is thought to help recruit telomerase to double-strand breaks [57], and it is possible that a similar interaction between Ku and the attached group II intron RNA contributes to the recruitment of Lig4 for some linear intron RNA retrohoming events. More generally, such a mechanism involving the interaction of Ku with RNA could also be used by LINE elements and other retrotransposons to recruit Lig4 and other NHEJ activities for cDNA integration and repair of DNA breaks.

Finally, our biochemical experiments demonstrate that template switching by the group II intron RT from the 5′ end of the intron RNA directly to the 3′ end of the upstream DNA exon is a potential alternate mechanism for cDNA attachment during linear intron RNA retrohoming. Although we found previously that the Ll.LtrB RT has low DNA-dependent DNA polymerase activity *in vitro* [21], we find here that it template switches and copies 5′-exon DNA templates as well as 5′-exon RNA templates (Figure 6), possibly reflecting that reverse transcription favors a conformation of the enzyme that can initiate more efficiently on DNA templates. In many cases, the template-switching junctions to DNA or RNA templates are seamless, but some have a small number of extra nucleotide residues, predominantly A-residues (corresponding to T-residues in the top strand) that were added by the RT to the 3′ end of the cDNA prior to the template switch. This pattern of extra nucleotide addition, which we found under both enzyme optimal and near-physiological conditions, differs from the majority of 5′-integration junctions resulting from linear intron retrohoming *in vivo*, where the extra nucleotide residues do not show a similar bias and sometimes correspond to copies of neighboring DNA sequences. It remains possible, however, that template switching by the Ll.LtrB RT could give different junctions *in vivo*, and that template switching and extra nucleotide addition by this enzyme contributes to some retrohoming events. We note that the ability of the group II intron RT's template switching activity to efficiently link sequences in two different templates could potentially be used to directly attach linker sequences containing primer-binding sites to the ends of cDNAs for cDNA cloning and sequencing applications.

## Materials and Methods

### *D. melanogaster* stocks

Flies were raised in standard fly media at 22°C. The *lig4^169^* mutant, obtained from Mitch McVey (Tufts University, Medford, MA), has a deletion that removes the start codon and most of the region encoding the ATPase and adenylation domains [31]. The *ku70^7B2^* and *ku70^Ex8^* mutants were obtained from William Engels (University of Wisconsin, Madison, WI). The *ku70^7B2^* allele lacks 1,359 bp at the 3′ end of the 2,393-bp gene, including most of the DNA and Ku80-interaction domains [43]. The *ku70^Ex8^* allele lacks at least 1 kb, including all of exon 1 and the start codon [43]. The *ku70^7B2^*/*ku70^Ex8^* genotype, generated by crosses between *trans*-heterozygous *ku70^7B2^*/*ku70^Ex8^* parents, is the same as that used previously to study double-strand break repair pathways [39], [43].

The *mus308^D2^* stock [60] was obtained from the *Drosophila* Stock Center (Bloomington, IA). The mutation lies outside of the coding region and results in undetectable levels of PolQ protein expression [38].

To obtain transgenic flies harboring a *lig4* rescue fragment, a 6-kb DNA segment containing the *lig4* gene was amplified from *w^1118^* genomic DNA by using the Expand High Fidelity PCR System (Roche Applied Science, Indianapolis, IN), with primers Lig4 F1 BamHI (5′-AAGAGGATCCAGTAGCTGTAGAAGCAGCCAAC) and Lig4 R1 XhoI 5′-AAGACTCGAGCAGCAGTTCCTCCGACATGAAG). This PCR product was inserted between BamHI and XhoI sites of the P-element transformation vector pCaSpeR4 [61], and transgenic flies were produced by GenetiVision (Houston, TX). A transgene insertion on chromosome 2 was recombined with *lig4^169^* to generate the fly stock used in *P{lig4^+^}* rescue experiments.

Except for the *trans*-heterozygous *ku70^7B2^*/*ku70^Ex8^* embryos (see above), embryos used for microinjection were obtained from crosses between isogenic wild-type or homozygous mutant parents. For all stocks, precellular blastoderm embryos were collected in egg laying chambers in under 40 min, microinjected with recipient plasmids and group II intron RNPs, and incubated for 1 h at 30°C prior to DNA extraction.

### Recombinant plasmids

pACD5C, which was used for synthesis of lariat and linear intron Ll.LtrB intron RNAs, is a derivative intron-donor plasmid pACD4C with a T7 promoter sequence inserted in the sense orientation at the SalI site in intron DIV [25], [62].

pBRR3-ltrB, the target plasmid for intron-integration assays, contains the Ll.LtrB intron homing site (ligated exon 1 and 2 sequences of the *ltrB* gene from positions −178 upstream to +91 downstream of the intron-insertion site) cloned upstream of a promoterless *tet^R^* gene in an Amp^R^ pBR322-based vector [63].

pIMP-1P, used for expression of the LtrA protein for RNP reconstitution, contains the LtrA ORF cloned downstream of a tac promoter and Φ10 Shine-Dalgarno sequence in the expression vector pCYB2 (New England BioLabs, Ipswich, MA) [9]. LtrA is expressed from this plasmid as a fusion protein with a C-terminal tag containing an intein-linked chitin-binding domain, enabling LtrA purification via a chitin-affinity column, followed by intein-cleavage [9].

pMAL-LtrA, used for expression of the LtrA protein for biochemical assays, contains the LtrA ORF [64] cloned downstream of a tac promoter and Φ10 Shine-Dalgarno sequence between BamHI and HindIII of the protein-expression vector pMAL-c2t. The latter is a derivative of pMal-c2x (New England BioLabs, Ipswich MA) with a TEV protease-cleavage site in place of the factor Xa site [65]. LtrA is expressed from this plasmid with an N-terminal fusion to maltose-binding protein (MalE), enabling its purification via an amylose-affinity column, followed by TEV-protease cleavage to remove the tag (see below).

### Preparation of Ll.LtrB lariat and linear RNAs

Ll.LtrB-ΔORF intron RNAs were transcribed from DNA templates generated by PCR of plasmid pACD5C with primers that append a phage T3 promoter sequence (underlined in sequences below) [26]. For the lariat precursor RNA, the PCR primers were pACD-T3 (5′-GGAGTCTAGAAATTAACCCTCACTAAAGGGAATTGTGAGCG) and NheIR (5′-CTAGCAGCACGCCATAGTGACTGGCG), and for linear intron RNA, the PCR primers were T3LIS-1G (5′-AATTAACCCTCACTAAAGTGCGCCCAGATAGGGTGTTAAGTCAAG) and HPLC-purified LtrB940a (5′-GTGAAGTAGGGAGGTACCGCCTTGTTC). The PCR products were purified by using the Wizard SV Gel and PCR Clean-up System (Promega), extracted with phenol-chloroform-isoamyl alcohol (phenol-CIA; 25∶24∶1 by volume), ethanol precipitated, and dissolved in nuclease-free water. *In vitro* transcription and the preparation of lariat and linear intron RNAs were as described [26].

### Preparation of LtrA protein and RNPs

The LtrA protein used for RNP reconstitution was expressed in *E. coli* BL21(DE3) from the intein-based expression vector pImp-1P and purified via a chitin-affinity column and intein cleavage, as described [9], except that the column buffer contained 50 mM Tris-HCl, pH 8.0, 0.1 mM EDTA, and 0.1% NP-40. Ll.LtrB RNPs were reconstituted with the purified LtrA protein and *in vitro*-synthesized lariat or linear Ll.LtrB-ΔORF intron RNA, as described [26], except that the final RNP pellet was dissolved in 10 mM KCl, 5 mM MgCl_2_, and 40 mM HEPES, pH 8.0.

The LtrA protein used in biochemical assays was expressed in *E. coli* BL21(DE3) from the plasmid pMAL-LtrA. Cells were grown in starter cultures of LB medium overnight at 37°C, inoculated into 0.5-l LB medium in ultra-yield flasks, and autoinduced by growing at 37°C for 3 h, followed by 18°C for 24 h [66]. Cells were harvested by centrifugation (Beckman JLA-8.1000; 4,000× g, 15 min, 4°C), resuspended in 1 M NaCl, 20 mM Tris-HCl, pH 7.5, 20% glycerol, and 0.1 mg/ml lysozyme (Sigma-Aldrich, St. Louis, MO), kept on ice for 15 min, and lysed by 3 freeze-thaw cycles on dry ice followed by sonication (Branson 450 Sonifier, Branson Ultrasonics, Danbury, CT; three or four 10 sec bursts on ice at an amplitude of 60%, with 10 sec between bursts). After pelleting cell debris (Beckman JA-14 rotor, 10,000 rpm, 30 min, 4°C), nucleic acids were precipitated from the supernatant with 0.4% polyethylenimine (PEI) and constant stirring for 20 min at 4°C, followed by centrifugation (Beckman JA-14 rotor, 14,000 rpm, 30 min, 4°C). Proteins were precipitated from the supernatant by adding ammonium sulfate to 50% saturation with constant stirring for 1 h at 4°C. The precipitated proteins were pelleted (Beckman JA-14 rotor, 14,000 rpm, 30 min, 4°C), dissolved in 500 mM NaCl, 20 mM Tris-HCl, pH 7.5, 10% glycerol, and run through a 10-ml amylose column (FPLC; Amylose High-Flow resin; New England BioLabs, Ipswich, MA), which was washed with 3 column volumes of 500 mM NaCl, 20 mM Tris-HCl, pH 7.5, 10% glycerol and eluted with 500 mM NaCl, 20 mM Tris-HCl, pH 7.5, 10% glycerol containing 10 mM maltose. Fractions containing the MalE-LtrA fusion were incubated with TEV protease (80 µg/ml, 18 h, at 4°C), and imidazole was added to a final concentration of 40 mM. LtrA freed of the MalE tag was then purified by FPLC through a Ni-NTA equilibrated with 500 mM NaCl, 20 mM Tris-HCl, pH 7.5, 10% glycerol, 40 mM imidazole. The Ni-NTA column, which takes advantage of endogenous histidine residues in LtrA's C-terminal domain, was washed with 3 column volumes of 500 mM NaCl, 20 mM Tris-HCl, pH 7.5, 10% glycerol, 40 mM imidazole, and eluted in 500 mM NaCl, 20 mM Tris-HCl, pH 7.5, 10% glycerol, 300 mM imidazole. Finally, the peak LtrA fractions from the Ni-NTA column were further purified through two tandem 1-ml heparin Sepharose columns (New England BioLabs). The columns were equilibrated with 500 mM NaCl, 20 mM Tris-HCl, pH 7.5, 10% glycerol, loaded directly with LtrA protein from the Ni-NTA column, washed with 5-column volumes of 500 mM NaCl, 20 mM Tris-HCl, pH 7.5, 10% glycerol, and eluted with a 20-column volume gradient of 0.5 to 1 M NaCl, 20 mM Tris-HCl, pH 7.5, 10% glycerol. The protein elutes approximately midway through the gradient at ∼750 mM NaCl. The purified protein was concentrated to 30 µM, exchanged into 100 mM NaCl, 20 mM Tris-HCl, pH 7.5, 10% glycerol by dialysis, flash-frozen in liquid nitrogen, and stored at −80°C.

### Retrohoming assays and analysis of intron-integration junctions

*Drosophila* embryos were microinjected with ∼300 pl recipient plasmid pBRR3-ltrB at 1.4 mg/ml in solution with 500 mM MgCl_2_ and 17 mM dNTPs, followed within 5 min by ∼300 pl of Ll.LtrB lariat or linear RNPs at 2.6 mg/ml in 10 mM KCl, 5 mM MgCl_2_, 40 mM HEPES, pH 8.0. The RNPs consist of LtrA protein bound to Ll.LtrB lariat or linear intron RNA with a phage T7 promoter sequence inserted in intron domain IV, and the recipient plasmid contains the Ll.LtrB intron target site (ligated exon 1 and 2 sequences of the *ltrB* gene; E1 and E2) cloned upstream of a promoterless *tet* ^R^ gene in a pBR322-based vector carrying an Amp^R^ marker. Site-specific integration of the intron into the target site introduces the T7 promoter upstream of the promoterless *tet* ^R^ gene, thereby activating that gene. Eighty embryos were injected and incubated at 30°C for 1 h for each assay. The pooled embryos were incubated in lysis buffer (20 mM Tris-HCl, pH 8.0, 5 mM EDTA, 400 mM NaCl, 1% SDS (w/v)) with 400 µg/ml proteinase K (Molecular Biology Grade; Sigma-Aldrich) for 1 h at 55°C, and then extracted with phenol-CIA. Nucleic acids were ethanol precipitated and dissolved in 12 µl of distilled water.

For assays of retrohoming efficiency, a 4-µl portion of the nucleic acid preparation was electroporated into electrocompetent *E. coli* HMS174(DE3) F^−^, *hsdR*, *recA*, *rif* ^r^ (Novagen, EMD Chemicals, Gibbstown, NJ), which expresses T7 RNA polymerase. Cells were plated at different dilutions on 2% agar containing LB medium with ampicillin (50 µg/ml) plus tetracycline (25 µg/ml) or the same concentration of ampicillin alone. Colonies were counted after overnight incubation at 37°C, and the integration efficiency was calculated as the ratio of (Amp^R^+Tet^R^)/Amp^R^ colonies.

For analysis of intron-integration junctions, a 1-µl portion of the nucleic acid preparation was used as template for PCR using Phusion High Fidelity PCR Master Mix with HF buffer (New England BioLabs). The 5′-junction PCRs were done with primers P1 (5′-CTGATCGATAGCTGAAACGC) and LtrB933a (5′-AGGGAGGTACCGCCTTGTTCACATTAC), and the 3′ junction PCRs were done with primers P3 (5′-CAGTGAATTTTTACGAACGAACAATAAC) and P4 (5′-AATGGACGATATCCCGCA). The PCR was done for 25 cycles for all strains, except for parallel assays of wild-type Or-R and the *polQ* mutant, which required 35 PCR cycles to obtain sufficient PCR product from the mutant. The PCR products were purified using a MinElute PCR purification Kit (Qiagen), cloned into a TOPO TA cloning vector (pCRII-TOPO; Invitrogen, Carlsbad, CA), and transformed into chemically competent *E. coli* (One Shot TOP10; Invitrogen). The cloned PCR products were then amplified from randomly picked colonies by colony PCR using Phusion High Fidelity PCR Master Mix with HF buffer and primers M13 F(-20) (5′- GTAAAACGACGGCCAGT) and M13 R(-26) (5′-CAGGAAACAGCTATGAC) for 25 cycles, and sequenced using primers M13 R(-24) (5′-GGAAACAGCTATGACCATG) or M13 F(-20) [67].

### Biochemical assays of group II intron RT template switching

Biochemical assays were done by incubating purified LtrA protein (40 nM) with synthetic oligonucleotide substrates that correspond to the 5′ end of the Ll.LtrB intron (60-nt Ll.LtrB RNA; 40 nM) with an annealed 5′-^32^P-labeled DNA primer corresponding to nascent cDNA (45-nt Pri c; 44 nM) in the presence of exon 1 RNA or DNA (40-nt E1; 40 nM) in 20 µl of reaction medium containing 450 mM NaCl, 5 mM MgCl_2_, 20 mM Tris-HCl, pH 7.5, 1 mM dithiothreitol (DTT) and 200 µM dNTPs. The reaction components were assembled on ice with substrate added last and then incubated at 30°C for 30 min. Reactions were terminated by phenol-CIA extraction. Portions of the reaction product (3 µl) were added to an equal volume of gel loading buffer II (95% formamide, 18 mM EDTA and 0.025% each of SDS, xylene cyanol, and bromophenol blue; Ambion, Austin, TX), denatured at 98°C for 7 min, and analyzed by electrophoresis in a denaturing 10 or 15% polyacrylamide gel, which was visualized by scanning with a PhosphorImager (Typhoon Trio, GE Healthcare, Piscataway, NJ). ^32^P-labeled DNA products were excised from the gel, amplified by PCR, as described (Sabine Mohr, Scott Kuersten, and A.M.L., manuscript in preparation), and cloned into the TOPO-TA pCR2.1 vector (Invitrogen), according to the manufacturer's protocol. Random colonies were picked and the cloned PCR products were amplified by colony PCR using Phusion High Fidelity PCR Master Mix/HF buffer with primers M13 F(-20) and M13 R(-26), and sequenced using the M13 R(-24) primer (see above).

The oligonucleotides used in the biochemical assays were Ll.LtrB RNA [LtrB5'S20Anchor6,5 RNA] ((5′- GUGCGCCCAGAUAGGGUGUUCUCGUUGGCAAUGGUGUCCAACUUGUGCUGCCAGUGCUCG), with an aminoblock on its 3′ end); annealed primer c (5′- CGAGCACTGGCAGCACAAG-deoxyuridine-TGGACACCATTGCCAACGAGAACAC); and exon 1 DNA (5′-TGTGATTGCAACCCACGTCGATCGTGAACACATCCATAAC) or RNA (5′-UGUGAUUGCAACCCACGUCGAUCGUGAACACAUCCAUAAC). Oligonucleotides complementary to exon 1 DNA or RNA were: exon 1 AS (5′-GTTATGGATGTGTTCACGATCGACGTGGGTTGCAATCACA) and exon 1 AS+9 (5′-AATGATATGGTTATGGATGTGTTCACGATCGACGTGGGTTGCAATCACA).

DNA and RNA oligonucleotides used in the assays were obtained from Integrated DNA Technologies (IDT; Coralville, IA) and purified in a denaturing 10% (w/v) polyacrylamide gel. DNA primers were 5′-end labeled with [γ-^32^P]-ATP (10 Ci/mmol; Perkin-Elmer, Waltham, MA) by using phage T4 polynucleotide kinase (New England BioLabs) according to the manufacturer's protocol. Complementary oligonucleotides were annealed at ratios of 1∶1 (E1 oligonucleotides) or 1∶1.1 Ll.LtrB/primer c by mixing at 20 times the final concentration in annealing buffer (100 mM Tris-HCl, pH 7.5, and 5 mM EDTA), heating to 82°C, and slowly cooling to 25°C for 45 min. The efficiency of annealing was assessed by electrophoresis in a non-denaturing 6% polyacrylamide gel containing Tris-borate-EDTA (90 mM Tris, 90 mM boric acid, 2 mM EDTA) at 30°C [67].

## Supporting Information

Figure S1 DNA sequences resulting from template switching of LtrA from Ll.LtrB RNA to another Ll.LtrB RNA. (A) and (B) Sequence of cDNA products resulting from template switching of the Ll.LtrB RT (LtrA protein) from the 5′ end of the initial Ll.LtrB RNA template/primer c substrate to second and third molecules of Ll.LtrB RNA. Some sequences appear to result from the use of primer c to initiate directly at or near the 3′ end of the Ll.LtrB RNA. Bands were excised from the gel, cloned, and sequenced, as described in Materials and Methods. The substrate and expected cDNA product sequences are shown boxed above each set of experimentally determined sequences. Extra or mutant nucleotide residues are shown in lower-case letters; microhomologies at ends prior to template switching are shown in parentheses; and dashes indicate absence of a nucleotide residue. Freq., frequency of occurrence; *, ^32^P-labeled at the 5′ end of primer c.
(TIF)

Figure S2 Template switching of the LtrA from Ll.LtrB RNA to exon 1 DNA or RNA at different salt concentrations. The Ll.LtrB intron RT (LtrA protein; 40 nM) was incubated with artificial substrates corresponding to the 5′ end of the Ll.LtrB intron (Ll.LtrB RNA; 40 nM) with an annealed 5′-^32^P-labeled DNA primer c (Pri c; 44 nM) in the presence of exon 1 (E1) DNA or RNA (40 nM; black and red, respectively), as diagrammed in schematics to the left of the gel. Reactions were done in media containing 200 µM dNTPs, 5 mM MgCl_2_, 20 mM Tris-HCl, pH 7.5, and 1 mM DTT plus 450 mM NaCl, 200 mM KCl, or 100 mM KCl for 30 min at 30°C. After terminating the reaction by phenol-CIA extraction, the products were analyzed in a denaturing 10% polyacrylamide gel. Lanes (1) and (2) ^32^P-labeled Pri c incubated without and with LtrA in 450 mM NaCl, respectively; (3–5) LtrA incubated with ^32^P-labeled Pri c and E1 DNA in 450 mM NaCl, 200 mM KCl, and 100 mM KCl, respectively; (6–8) LtrA incubated with Ll.LtrB RNA with annealed ^32^P-labeled Pri c and E1 RNA in 450 mM NaCl, 200 mM KCl, and 100 mM KCl, respectively. Bands excised for sequencing are indicated in the gel. In the schematics, DNA and RNA oligonucleotides are shown in black and red, respectively; LtrA is shown as a gray oval; and the direction of DNA synthesis is indicated by a green arrow. The numbers to the right of the gel indicate the positions of 5′-end labeled size markers (10-bp DNA ladder, Invitrogen).
(TIF)

Figure S3 DNA sequences from template switching from Ll.LtrB RNA to exon 1 DNA or RNA under near physiological conditions. Sequences of DNAs resulting from template switching of the Ll.LtrB RT (LtrA) from the 5′ end of the Ll.LtrB RNA template/primer c DNA substrate to exon 1 DNA or RNA in reaction medium containing 100 mM KCl and 5 mM MgCl_2_ (Figure S2; lanes 5 and 8, respectively). Bands were excised from the gel, cloned, and sequenced, as described in Materials and Methods. The substrate and expected cDNA or DNA product sequences are shown boxed above each set of experimentally determined sequences. Extra or mutant nucleotide residues are shown in lower-case letters, and dashes indicate absence of a nucleotide residue. Freq., frequency of occurrence; *, ^32^P-label at 5′ end of primer c.
(TIF)

Figure S4 Non-denaturing gel analysis of annealed oligonucleotides used in 5′ and 3′-intron integration assays. 5′-^32^P-labeled oligonucleotides by themselves or annealed to a complementary DNA strand (see Materials and Methods), were diluted 1∶20 into 450 mM NaCl, 5 mM MgCl_2_, 20 mM Tris-HCl, pH 7.5 and incubated for 30 min at 30°C. The samples were then mixed 6∶1 with 30°C non-denaturing loading buffer (0.25% bromophenol blue, 0.25% xylene cyanol and 1.5% Ficoll 400 and analyzed by electrophoresis in a non-denaturing 6% polyacrylamide gel containing Tris-borate-EDTA (90 mM Tris, 90 mM boric acid, 2 mM EDTA) at 30°C [67]. Gels were soaked for 15 min in 25% isopropanol, 20% glycerol and 10% acetic acid to prevent cracking during drying, dried, and scanned with a PhosphorImager (Typhoon Trio, GE Healthcare). In the schematics below the gel, DNA and RNA oligonucleotides are shown in black and red, respectively. Lanes (1) 40 nM ^32^P-labeled Ll.LtrB RNA; (2) 40 nM ^32^P-labeled Ll.LtrB RNA annealed with 44 nM DNA primer c (Pri c); (3) 40 nM ^32^P-labeled exon 1 (E1) DNA; (4) 40 nM ^32^P-labeled E1 DNA annealed with 40 nM E1 AS DNA; (5) 40 nM ^32^P-labeled E1 RNA; (6) 40 nM ^32^P-labeled E1 RNA annealed with 40 nM E1 AS DNA; (7) 40 nM ^32^P-labeled E1 AS DNA; (8) 40 nM ^32^P-labeled E1 AS DNA annealed with 40 nM E1 DNA; (9) 40 nM ^32^P-labeled E1 AS+9 DNA; (10) 40 nM ^32^P-labeled E1 AS+9 DNA annealed with 40 nM E1 DNA; (11) 40 nM ^32^P-labeled E1 DNA; (12) 40 nM ^32^P-labeled E1 DNA annealed with 40 nM E1 AS+9 DNA.
(TIF)

Figure S5 DNA sequences of additional products obtained in template-switching experiments to double-strand E1 DNA with a 9-nt 5′-overhang. The figure shows sequences of additional products from bands o and p of Figure 6 lane 9 that result from using primer c to initiate directly on the exon 1 AS+9 DNA or at or near the 3′ end of Ll.LtrB RNA. One product (bottom) results from multiple template switches to exon 1 AS+9 DNA and Ll.LtrB RNA. Bands were excised from the gel, cloned, and sequenced, as described in Materials and Methods. The substrate and expected cDNA or DNA product sequences (boxed) are shown above each set of experimentally determined DNA sequences. Extra or mutant nucleotide residues are shown in lower-case letters, and dashes indicate absence of a nucleotide residue.
(TIF)

Table S1 Summary of experiments comparing retrohoming efficiencies of linear and lariat Ll.LtrB intron RNAs in wild-type and mutant *D. melanogaster* embryos. Retrohoming assays using lariat and linear RNPs were done in *D. melanogaster* precellular blastoderm embryos, as described in Figure 2A and Materials and Methods. After incubating the embryos for 1 h at 30°C, nucleic acids were extracted and transformed into *E. coli* HMS174(DE3) for plating assays of retrohoming efficiency. WT, wild type. ^a^Retrohoming efficiency calculated as (Tet^R^+Amp^R^)/Amp^R^ colonies. ^b^Retrohoming efficiency relative to wild-type *w^1118^* or Or-R assayed in parallel.
(DOC)

## References

[1] Lambowitz AM, Zimmerly S, Gestland RF, Cech TR, Atkins JF (2011). Group II introns: mobile ribozymes that invade DNA.. RNA worlds: from life's origins to diversity in gene regulation.

[2] Keating KS, Toor N, Perlman PS, Pyle AM (2010). A structural analysis of the group II intron active site and implications for the spliceosome.. RNA.

[3] Martin W, Koonin EV (2006). Introns and the origin of nucleus-cytosol compartmentalization.. Nature.

[4] Rodríguez-Trelles F, Tarrío R, Ayala FJ (2006). Origins and evolution of spliceosomal introns.. Annu Rev Genet.

[5] Peebles CL, Perlman PS, Mecklenburg KL, Petrillo ML, Tabor JH (1986). A self-splicing RNA excises an intron lariat.. Cell.

[6] Pyle AM, Lambowitz AM, Gesteland RF, Cech T, Atkins JF (2006). Group II introns: ribozymes that splice RNA and invade DNA.. The RNA World, third edition.

[7] Carignani G, Groudinsky O, Frezza D, Schiavon E, Bergantino E (1983). An mRNA maturase is encoded by the first intron of the mitochondrial gene for the subunit I of cytochrome oxidase in S. cerevisiae.. Cell.

[8] Matsuura M, Noah JW, Lambowitz AM (2001). Mechanism of maturase-promoted group II intron splicing.. EMBO J.

[9] Saldanha R, Chen B, Wank H, Matsuura M, Edwards J (1999). RNA and protein catalysis in group II intron splicing and mobility reactions using purified components.. Biochemistry.

[10] Yang J, Zimmerly S, Perlman PS, Lambowitz AM (1996). Efficient integration of an intron RNA into double-stranded DNA by reverse splicing.. Nature.

[11] Cousineau B, Smith D, Lawrence-Cavanagh S, Mueller JE, Yang J (1998). Retrohoming of a bacterial group II intron: mobility via complete reverse splicing, independent of homologous DNA recombination.. Cell.

[12] Dickson L, Huang HR, Liu L, Matsuura M, Lambowitz AM (2001). Retrotransposition of a yeast group II intron occurs by reverse splicing directly into ectopic DNA sites.. Proc Natl Acad Sci U S A.

[13] Eskes R, Yang J, Lambowitz AM, Perlman PS (1997). Mobility of yeast mitochondrial group II introns: engineering a new site specificity and retrohoming via full reverse splicing.. Cell.

[14] Ichiyanagi K, Beauregard A, Lawrence S, Smith D, Cousineau B (2002). Retrotransposition of the Ll.LtrB group II intron proceeds predominantly via reverse splicing into DNA targets.. Mol Microbiol.

[15] Schmelzer C, Schweyen RJ (1986). Self-splicing of group II introns in vitro: mapping of the branch point and mutational inhibition of lariat formation.. Cell.

[16] van der Veen R, Kwakman JH, Grivell LA (1987). Mutations at the lariat acceptor site allow self-splicing of a group II intron without lariat formation.. EMBO J.

[17] Podar M, Chu VT, Pyle AM, Perlman PS (1998). Group II intron splicing *in vivo* by first-step hydrolysis.. Nature.

[18] Li CF, Costa M, Bassi G, Lai YK, Michel F (2011). Recurrent insertion of 5′-terminal nucleotides and loss of the branchpoint motif in lineages of group II introns inserted in mitochondrial preribosomal RNAs.. RNA.

[19] Vogel J, Borner T (2002). Lariat formation and a hydrolytic pathway in plant chloroplast group II intron splicing.. EMBO J.

[20] Green MR (1986). Pre-mRNA splicing.. Annu Rev Genet.

[21] Smith D, Zhong J, Matsuura M, Lambowitz AM, Belfort M (2005). Recruitment of host functions suggests a repair pathway for late steps in group II intron retrohoming.. Genes Dev.

[22] Martínez-Abarca F, Barrientos-Durán A, Fernández-López M, Toro N (2004). The RmInt1 group II intron has two different retrohoming pathways for mobility using predominantly the nascent lagging strand at DNA replication forks for priming.. Nucleic Acids Res.

[23] Zhong J, Lambowitz AM (2003). Group II intron mobility using nascent strands at DNA replication forks to prime reverse transcription.. EMBO J.

[24] Eskes R, Liu L, Ma H, Chao MY, Dickson L (2000). Multiple homing pathways used by yeast mitochondrial group II introns.. Mol Cell Biol.

[25] Zhuang F, Mastroianni M, White TB, Lambowitz AM (2009). Linear group II intron RNAs can retrohome in eukaryotes and may use nonhomologous end-joining for cDNA ligation.. Proc Natl Acad Sci U S A.

[26] Mastroianni M, Watanabe K, White TB, Zhuang F, Vernon J (2008). Group II intron-based gene targeting reactions in eukaryotes.. PLoS ONE.

[27] Mörl M, Niemer I, Schmelzer C (1992). New reactions catalyzed by a group II intron ribozyme with RNA and DNA substrates.. Cell.

[28] Adams MD, McVey M, Sekelsky JJ (2003). Drosophila BLM in double-strand break repair by synthesis-dependent strand annealing.. Science.

[29] Hagmann M, Adlkofer K, Pfeiffer P, Bruggmann R, Georgiev O (1996). Dramatic changes in the ratio of homologous recombination to nonhomologous DNA-end joining in oocytes and early embryos of *Xenopus laevis*.. Biol Chem Hoppe Seyler.

[30] Hagmann M, Bruggmann R, Xue L, Georgiev O, Schaffner W (1998). Homologous recombination and DNA-end joining reactions in zygotes and early embryos of zebrafish (*Danio rerio*) and *Drosophila melanogaster*.. Biol Chem.

[31] McVey M, Radut D, Sekelsky JJ (2004). End-joining repair of double-strand breaks in *Drosophila melanogaster* is largely DNA ligase IV independent.. Genetics.

[32] Beauregard A, Curcio MJ, Belfort M (2008). The take and give between retrotransposable elements and their hosts.. Annu Rev Genet.

[33] Suzuki J, Yamaguchi K, Kajikawa M, Ichiyanagi K, Adachi N (2009). Genetic evidence that the non-homologous end-joining repair pathway is involved in LINE retrotransposition.. PLoS Genet.

[34] Sawyer SL, Malik HS (2006). Positive selection of yeast nonhomologous end-joining genes and a retrotransposon conflict hypothesis.. Proc Natl Acad Sci U S A.

[35] Bibillo A, Eickbush TH (2002). The reverse transcriptase of the R2 non-LTR retrotransposon: continuous synthesis of cDNA on non-continuous RNA templates.. J Mol Biol.

[36] Chen B, Lambowitz AM (1997). *De novo* and DNA primer-mediated initiation of cDNA synthesis by the Mauriceville retroplasmid reverse transcriptase involve recognition of a 3′ CCA sequence.. J Mol Biol.

[37] Kennell JC, Wang H, Lambowitz AM (1994). The Mauriceville plasmid of *Neurospora* spp. uses novel mechanisms for initiating reverse transcription *in vivo*.. Mol Cell Biol.

[38] Chan SH, Yu AM, McVey M (2010). Dual roles for DNA polymerase theta in alternative end-joining repair of double-strand breaks in *Drosophila*.. PLoS Genet.

[39] Yu AM, McVey M (2010). Synthesis-dependent microhomology-mediated end joining accounts for multiple types of repair junctions.. Nucleic Acids Res.

[40] Guo H, Karberg M, Long M, Jones JP, Sullenger B (2000). Group II introns designed to insert into therapeutically relevant DNA target sites in human cells.. Science.

[41] Adachi N, Ishino T, Ishii Y, Takeda S, Koyama H (2001). DNA ligase IV-deficient cells are more resistant to ionizing radiation in the absence of Ku70: Implications for DNA double-strand break repair.. Proc Natl Acad Sci U S A.

[42] Nick McElhinny SA, Snowden CM, McCarville J, Ramsden DA (2000). Ku recruits the XRCC4-ligase IV complex to DNA ends.. Mol Cell Biol.

[43] Johnson-Schlitz DM, Flores C, Engels WR (2007). Multiple-pathway analysis of double-strand break repair mutations in *Drosophila*.. PLoS Genet.

[44] Bibillo A, Eickbush TH (2004). End-to-end template jumping by the reverse transcriptase encoded by the R2 retrotransposon.. J Biol Chem.

[45] Clark JM, Joyce CM, Beardsley GP (1987). Novel blunt-end addition reactions catalyzed by DNA polymerase I of *Escherichia coli*.. J Mol Biol.

[46] Clark JM (1988). Novel non-templated nucleotide addition reactions catalyzed by procaryotic and eucaryotic DNA polymerases.. Nucleic Acids Res.

[47] Golinelli MP, Hughes SH (2002). Nontemplated base addition by HIV-1 RT can induce nonspecific strand transfer in vitro.. Virology.

[48] Hu G (1993). DNA polymerase-catalyzed addition of nontemplated extra nucleotides to the 3′ end of a DNA fragment.. DNA Cell Biol.

[49] Bowater R, Doherty AJ (2006). Making ends meet: repairing breaks in bacterial DNA by non-homologous end-joining.. PLoS Genet.

[50] Gilbert N, Lutz S, Morrish TA, Moran JV (2005). Multiple fates of L1 retrotransposition intermediates in cultured human cells.. Mol Cell Biol.

[51] Morrish TA, Gilbert N, Myers JS, Vincent BJ, Stamato TD (2002). DNA repair mediated by endonuclease-independent LINE-1 retrotransposition.. Nat Genet.

[52] Symer DE, Connelly C, Szak ST, Caputo EM, Cost GJ (2002). Human l1 retrotransposition is associated with genetic instability in vivo.. Cell.

[53] Liang F, Romanienko PJ, Weaver DT, Jeggo PA, Jasin M (1996). Chromosomal double-strand break repair in Ku80-deficient cells.. Proc Natl Acad Sci U S A.

[54] Ma JL, Kim EM, Haber JE, Lee SE (2003). Yeast Mre11 and Rad1 proteins define a Ku-independent mechanism to repair double-strand breaks lacking overlapping end sequences.. Mol Cell Biol.

[55] Yu X, Gabriel A (2003). Ku-dependent and Ku-independent end-joining pathways lead to chromosomal rearrangements during double-strand break repair in *Saccharomyces cerevisiae*.. Genetics.

[56] Peterson SE, Stellwagen AE, Diede SJ, Singer MS, Haimberger ZW (2001). The function of a stem-loop in telomerase RNA is linked to the DNA repair protein Ku.. Nat Genet.

[57] Stellwagen AE, Haimberger ZW, Veatch JR, Gottschling DE (2003). Ku interacts with telomerase RNA to promote telomere addition at native and broken chromosome ends.. Genes Dev.

[58] Ting NS, Yu Y, Pohorelic B, Lees-Miller SP, Beattie TL (2005). Human Ku70/80 interacts directly with hTR, the RNA component of human telomerase.. Nucleic Acids Res.

[59] Dickson L, Connell S, Huang HR, Henke RM, Liu L (2004). Abortive transposition by a group II intron in yeast mitochondria.. Genetics.

[60] Boyd JB, Sakaguchi K, Harris PV (1990). mus308 mutants of *Drosophila* exhibit hypersensitivity to DNA cross-linking agents and are defective in a deoxyribonuclease.. Genetics.

[61] Thummel CS (1992). Mechanisms of transcriptional timing in *Drosophila*.. Science.

[62] Perutka J, Wang W, Goerlitz D, Lambowitz AM (2004). Use of computer-designed group II introns to disrupt *Escherichia coli* DExH/D-box protein and DNA helicase genes.. J Mol Biol.

[63] Karberg M, Guo H, Zhong J, Coon R, Perutka J (2001). Group II introns as controllable gene targeting vectors for genetic manipulation of bacteria.. Nat Biotechnol.

[64] Mills DA, McKay LL, Dunny GM (1996). Splicing of a group II intron involved in the conjugative transfer of pRS01 in lactococci.. J Bacteriol.

[65] Kristelly R, Earnest BT, Krishnamoorthy L, Tesmer JJ (2003). Preliminary structure analysis of the DH/PH domains of leukemia-associated RhoGEF.. Acta Crystallogr D Biol Crystallogr.

[66] Studier FW (2005). Protein production by auto-induction in high density shaking cultures.. Protein Expr Purif.

[67] Sambrook J, Fritsch EF, Maniatis T (1989). Molecular Cloning: A Laboratory Manual.

