# Supplementary figures and images for: The Retrohoming of Linear Group II Intron RNAs in Drosophila melanogaster Occurs by Both DNA Ligase 4–Dependent and –Independent Mechanisms
Source: PLoS Genet. 2012 Feb 16;8(2):e1002534. doi: 10.1371/journal.pgen.1002534 (PMC3280974; doi:10.1371/journal.pgen.1002534)

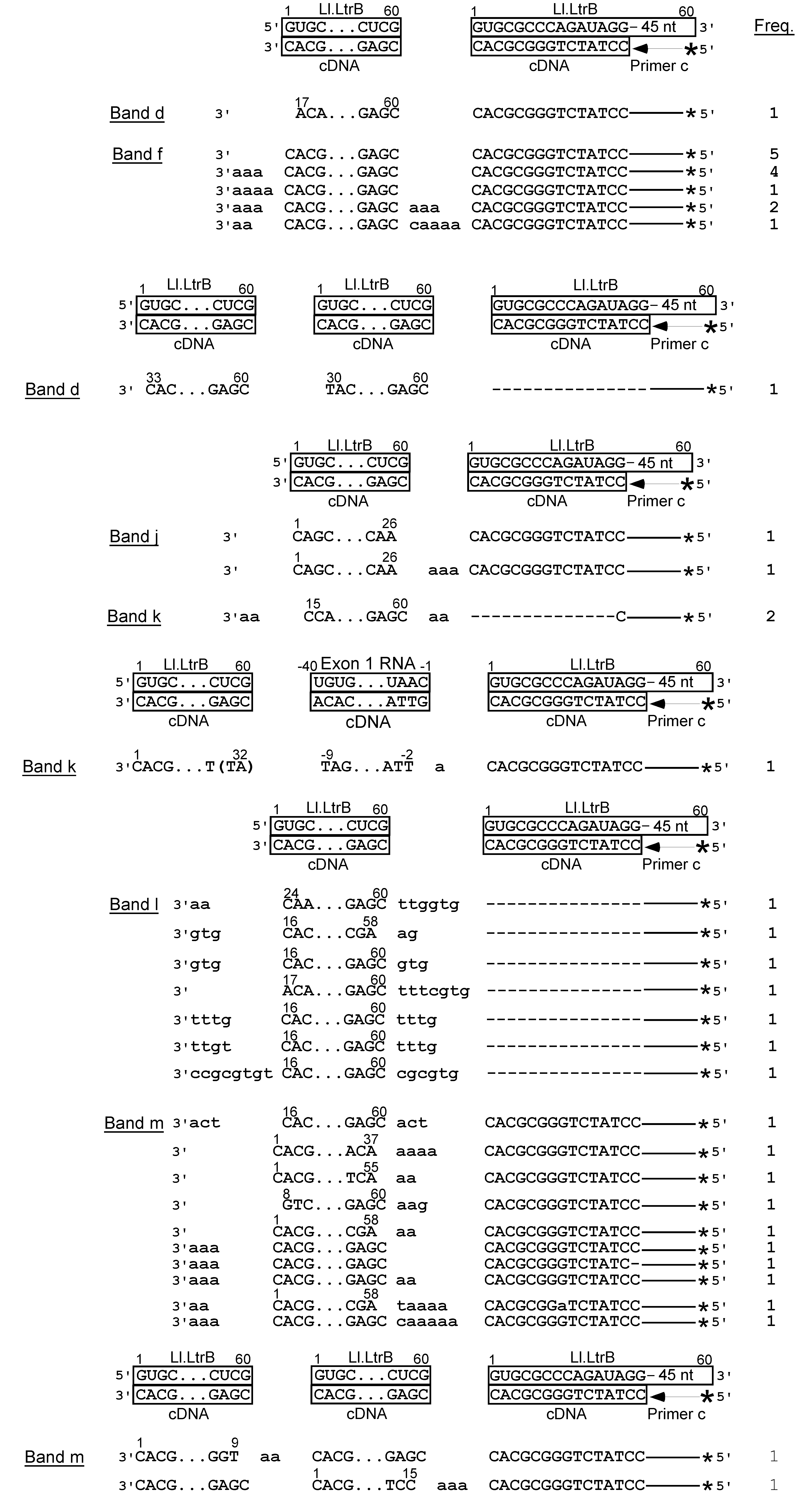

Supplement: Figure S1 — DNA sequences resulting from template switching of LtrA from Ll.LtrB RNA to another Ll.LtrB RNA. (A) and (B) Sequence of cDNA products resulting from template switching of the Ll.LtrB RT (LtrA protein) from the 5′ end of the initial Ll.LtrB RNA template/primer c substrate to second and third molecules of Ll.LtrB RNA. Some sequences appear to result from the use of primer c to initiate directly at or near the 3′ end of the Ll.LtrB RNA. Bands were excised from the gel, cloned, and sequenced, as described in Materials and Methods. The substrate and expected cDNA product sequences are shown boxed above each set of experimentally determined sequences. Extra or mutant nucleotide residues are shown in lower-case letters; microhomologies at ends prior to template switching are shown in parentheses; and dashes indicate absence of a nucleotide residue. Freq., frequency of occurrence; *, 32P-labeled at the 5′ end of primer c. (TIF) [file pgen.1002534.s001.tif]

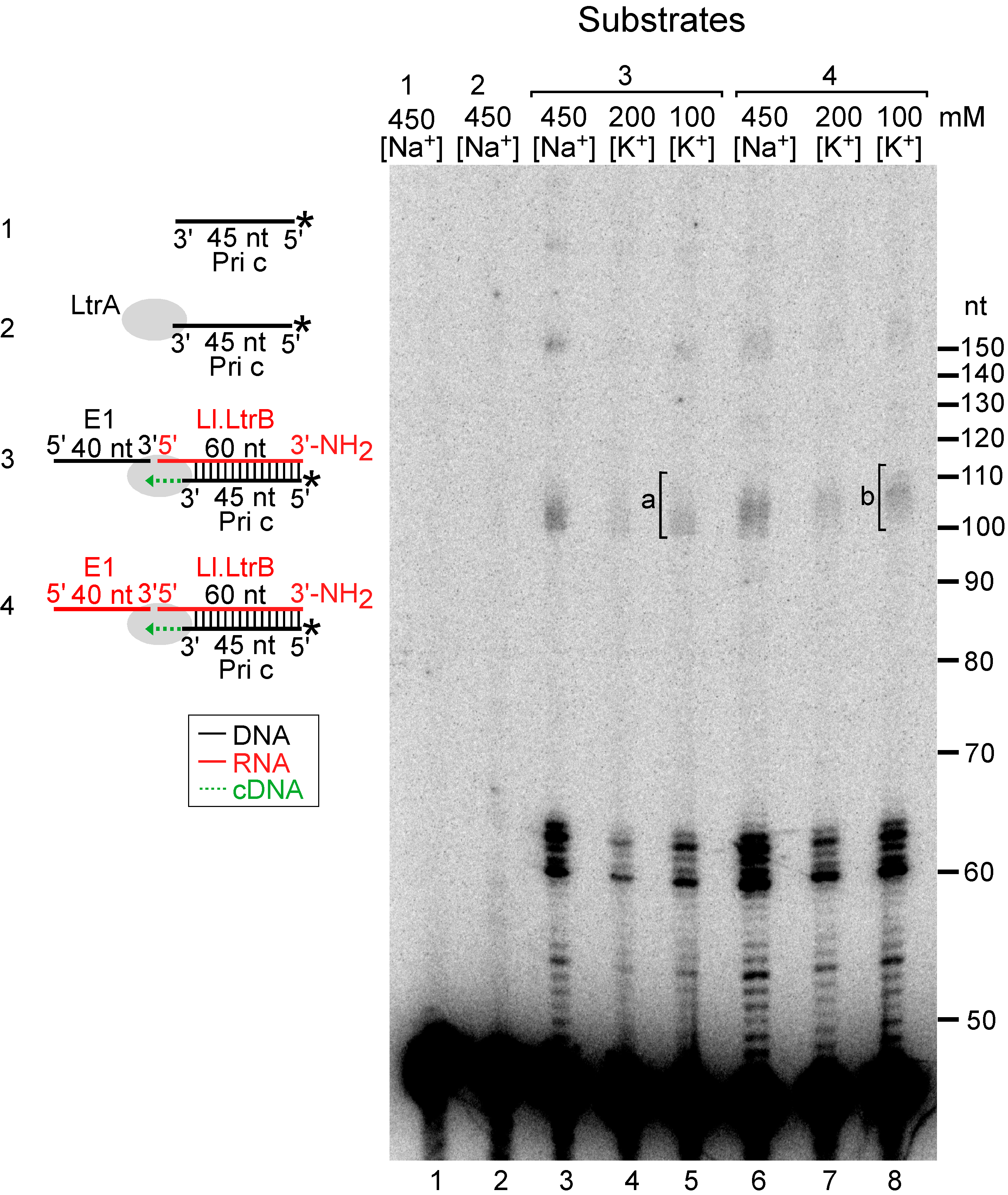

Supplement: Figure S2 — Template switching of the LtrA from Ll.LtrB RNA to exon 1 DNA or RNA at different salt concentrations. The Ll.LtrB intron RT (LtrA protein; 40 nM) was incubated with artificial substrates corresponding to the 5′ end of the Ll.LtrB intron (Ll.LtrB RNA; 40 nM) with an annealed 5′-32P-labeled DNA primer c (Pri c; 44 nM) in the presence of exon 1 (E1) DNA or RNA (40 nM; black and red, respectively), as diagrammed in schematics to the left of the gel. Reactions were done in media containing 200 µM dNTPs, 5 mM MgCl2, 20 mM Tris-HCl, pH 7.5, and 1 mM DTT plus 450 mM NaCl, 200 mM KCl, or 100 mM KCl for 30 min at 30°C. After terminating the reaction by phenol-CIA extraction, the products were analyzed in a denaturing 10% polyacrylamide gel. Lanes (1) and (2) 32P-labeled Pri c incubated without and with LtrA in 450 mM NaCl, respectively; (3–5) LtrA incubated with 32P-labeled Pri c and E1 DNA in 450 mM NaCl, 200 mM KCl, and 100 mM KCl, respectively; (6–8) LtrA incubated with Ll.LtrB RNA with annealed 32P-labeled Pri c and E1 RNA in 450 mM NaCl, 200 mM KCl, and 100 mM KCl, respectively. Bands excised for sequencing are indicated in the gel. In the schematics, DNA and RNA oligonucleotides are shown in black and red, respectively; LtrA is shown as a gray oval; and the direction of DNA synthesis is indicated by a green arrow. The numbers to the right of the gel indicate the positions of 5′-end labeled size markers (10-bp DNA ladder, Invitrogen). (TIF) [file pgen.1002534.s002.tif]

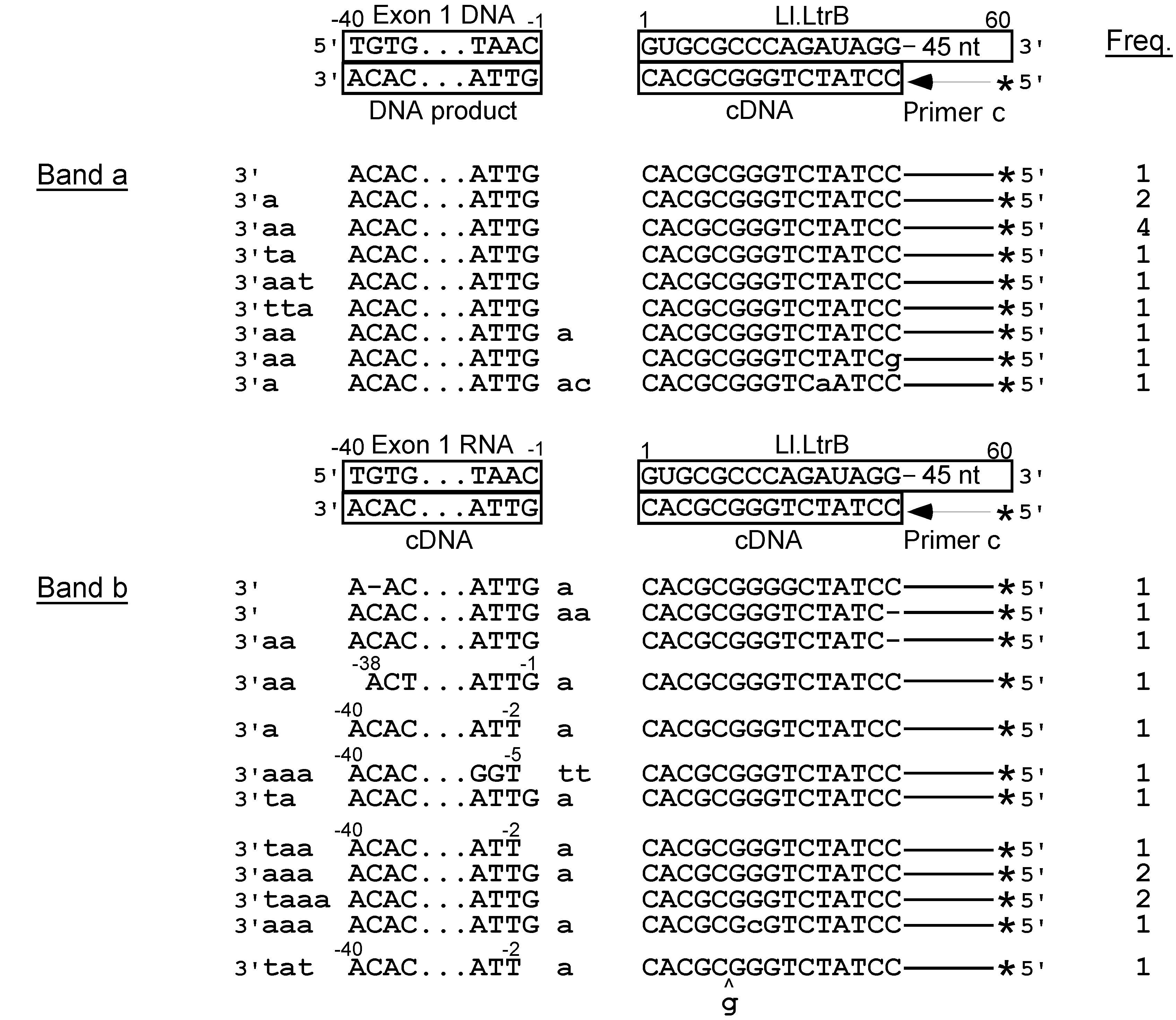

Supplement: Figure S3 — DNA sequences from template switching from Ll.LtrB RNA to exon 1 DNA or RNA under near physiological conditions. Sequences of DNAs resulting from template switching of the Ll.LtrB RT (LtrA) from the 5′ end of the Ll.LtrB RNA template/primer c DNA substrate to exon 1 DNA or RNA in reaction medium containing 100 mM KCl and 5 mM MgCl2 (Figure S2; lanes 5 and 8, respectively). Bands were excised from the gel, cloned, and sequenced, as described in Materials and Methods. The substrate and expected cDNA or DNA product sequences are shown boxed above each set of experimentally determined sequences. Extra or mutant nucleotide residues are shown in lower-case letters, and dashes indicate absence of a nucleotide residue. Freq., frequency of occurrence; *, 32P-label at 5′ end of primer c. (TIF) [file pgen.1002534.s003.tif]

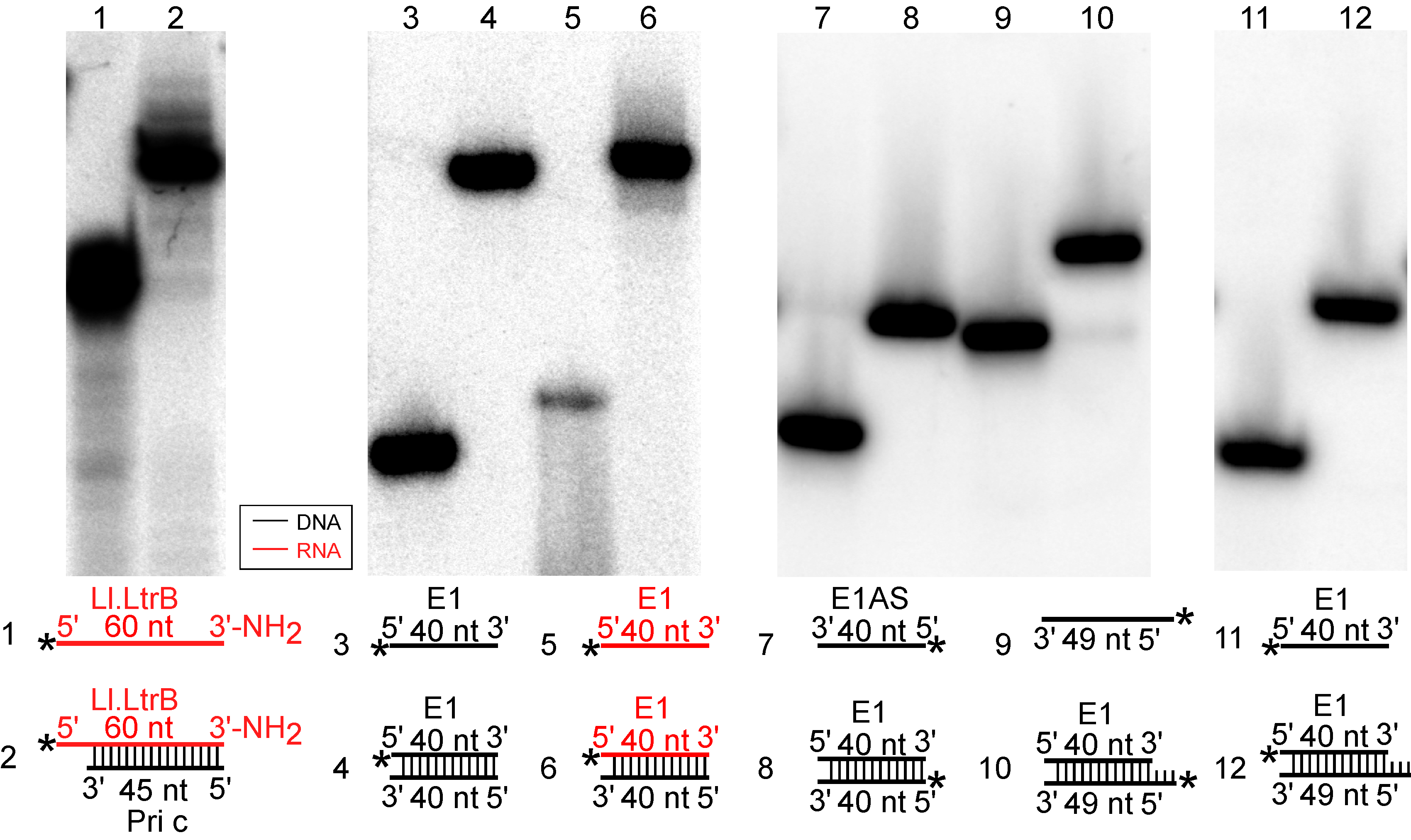

Supplement: Figure S4 — Non-denaturing gel analysis of annealed oligonucleotides used in 5′ and 3′-intron integration assays. 5′-32P-labeled oligonucleotides by themselves or annealed to a complementary DNA strand (see Materials and Methods), were diluted 1∶20 into 450 mM NaCl, 5 mM MgCl2, 20 mM Tris-HCl, pH 7.5 and incubated for 30 min at 30°C. The samples were then mixed 6∶1 with 30°C non-denaturing loading buffer (0.25% bromophenol blue, 0.25% xylene cyanol and 1.5% Ficoll 400 and analyzed by electrophoresis in a non-denaturing 6% polyacrylamide gel containing Tris-borate-EDTA (90 mM Tris, 90 mM boric acid, 2 mM EDTA) at 30°C [67]. Gels were soaked for 15 min in 25% isopropanol, 20% glycerol and 10% acetic acid to prevent cracking during drying, dried, and scanned with a PhosphorImager (Typhoon Trio, GE Healthcare). In the schematics below the gel, DNA and RNA oligonucleotides are shown in black and red, respectively. Lanes (1) 40 nM 32P-labeled Ll.LtrB RNA; (2) 40 nM 32P-labeled Ll.LtrB RNA annealed with 44 nM DNA primer c (Pri c); (3) 40 nM 32P-labeled exon 1 (E1) DNA; (4) 40 nM 32P-labeled E1 DNA annealed with 40 nM E1 AS DNA; (5) 40 nM 32P-labeled E1 RNA; (6) 40 nM 32P-labeled E1 RNA annealed with 40 nM E1 AS DNA; (7) 40 nM 32P-labeled E1 AS DNA; (8) 40 nM 32P-labeled E1 AS DNA annealed with 40 nM E1 DNA; (9) 40 nM 32P-labeled E1 AS+9 DNA; (10) 40 nM 32P-labeled E1 AS+9 DNA annealed with 40 nM E1 DNA; (11) 40 nM 32P-labeled E1 DNA; (12) 40 nM 32P-labeled E1 DNA annealed with 40 nM E1 AS+9 DNA. (TIF) [file pgen.1002534.s004.tif]

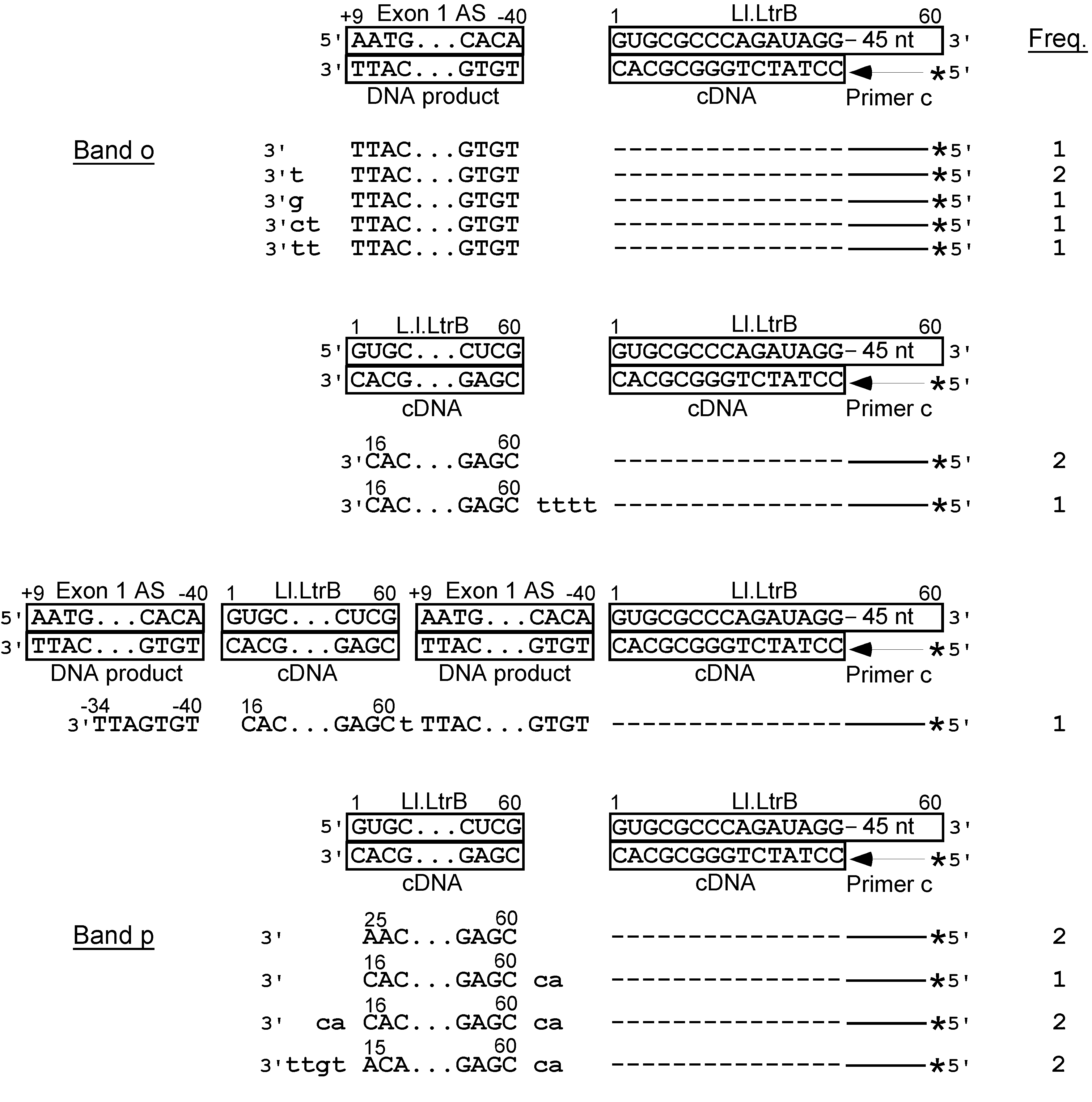

Supplement: Figure S5 — DNA sequences of additional products obtained in template-switching experiments to double-strand E1 DNA with a 9-nt 5′-overhang. The figure shows sequences of additional products from bands o and p of Figure 6 lane 9 that result from using primer c to initiate directly on the exon 1 AS+9 DNA or at or near the 3′ end of Ll.LtrB RNA. One product (bottom) results from multiple template switches to exon 1 AS+9 DNA and Ll.LtrB RNA. Bands were excised from the gel, cloned, and sequenced, as described in Materials and Methods. The substrate and expected cDNA or DNA product sequences (boxed) are shown above each set of experimentally determined DNA sequences. Extra or mutant nucleotide residues are shown in lower-case letters, and dashes indicate absence of a nucleotide residue. (TIF) [file pgen.1002534.s005.tif]
