# Supplementary material for: The Retrohoming of Linear Group II Intron RNAs in Drosophila melanogaster Occurs by Both DNA Ligase 4–Dependent and –Independent Mechanisms
Source: PLoS Genet. 2012 Feb 16;8(2):e1002534. doi: 10.1371/journal.pgen.1002534 (PMC3280974; doi:10.1371/journal.pgen.1002534)
Supplement: Table S1 — Summary of experiments comparing retrohoming efficiencies of linear and lariat Ll.LtrB intron RNAs in wild-type and mutant D. melanogaster embryos. Retrohoming assays using lariat and linear RNPs were done in D. melanogaster precellular blastoderm embryos, as described in Figure 2A and Materials and Methods. After incubating the embryos for 1 h at 30°C, nucleic acids were extracted and transformed into E. coli HMS174(DE3) for plating assays of retrohoming efficiency. WT, wild type. aRetrohoming efficiency calculated as (TetR+AmpR)/AmpR colonies. bRetrohoming efficiency relative to wild-type w1118 or Or-R assayed in parallel. (DOC) [file pgen.1002534.s006.doc]

**Table S1. Summary of experiments comparing retrohoming efficiencies of linear and lariat Ll.LtrB intron RNAs in wild-type and mutant *D. melanogaster* embryos.**

| Experiment | Stock | Intron | Retrohoming efficiency^a^ (%) | Retrohoming efficiency^b^ (% WT) |
| --- | --- | --- | --- | --- |
| 1 | Or-R (WT) | Linear | 0.11 |  |
|  | *lig4^-^* | Linear | 0.047 | 43 |
|  | Or-R (WT) | Lariat | 3.2 |  |
|  | *lig4^-^* | Lariat | 3.7 | 116 |
| 2 | Or-R (WT) | Linear | 0.042 |  |
|  | *lig4^-^* | Linear | 0.005 | 12 |
|  | Or-R (WT) | Lariat | 2.4 |  |
|  | *lig4*^-^ | Lariat | 3.3 | 138 |
| 3 | *w^1118^* (WT) | Linear | 0.081 |  |
|  | *ku70^-^* | Linear | 0.052 | 64 |
|  | *lig4^-^* | Linear | 0.021 | 25 |
|  | *w^1118^* (WT) | Lariat | 2.3 |  |
|  | *ku70^-^* | Lariat | 1.8 | 78 |
|  | *lig4^-^* | Lariat | 2.0 | 86 |
| 4 | Or-R (WT) | Linear | 0.14 |  |
|  | *ku70^-^* | Linear | 0.048 | 34 |
|  | *lig4^-^* | Linear | 0.012 | 9 |
|  | Or-R (WT) | Lariat | 3.5 |  |
|  | *ku70^-^* | Lariat | 3.5 | 100 |
|  | *lig4^-^* | Lariat | 2.5 | 71 |
| 5 | *w^1118^* (WT) | Linear | 0.023 |  |
|  | *ku70^-^* | Linear | 0.018 | 78 |
|  | *lig4^-^* | Linear | 0.0002 | 9 |
|  | *lig4^-^*; *P{lig4^+^}* | Linear | 0.034 | 148 |
|  | *w^1118^* (WT) | Lariat | 2.8 |  |
|  | *ku70^-^* | Lariat | 1.4 | 50 |
|  | *lig4^-^* | Lariat | 3.7 | 132 |
|  | *lig4^-^*; *P{lig4^+^}* | Lariat | 3.4 | 121 |

| Experiment | Stock | Intron | Retrohoming efficiency^a^ (%) | Retrohoming efficiency^b^ (%WT) |
| --- | --- | --- | --- | --- |
| 6 | *w^1118^* (WT) | Linear | 0.071 |  |
|  | *ku70^-^* | Linear | 0.0027 | 4 |
|  | *lig4^-^* | Linear | 0.0051 | 7 |
|  | *lig4^-^*; *P{lig4^+^}* | Linear | 0.06 | 86 |
|  | *w^1118^* (WT) | Lariat | 2.5 |  |
|  | *ku70^-^* | Lariat | 0.9 | 36 |
|  | *lig4^-^* | Lariat | 2.8 | 112 |
|  | *lig4^-^*; *P{lig4^+^}* | Lariat | 1.3 | 52 |
| 7 | *w^1118^* (WT) | Linear | 0.06 |  |
|  | *ku70^-^* | Linear | 0.031 | 52 |
|  | *lig4^-^* | Linear | 0.012 | 20 |
|  | *lig4^-^*; *P{lig4^+^}* | Linear | 0.051 | 86 |
|  | *w^1118^* (WT) | Lariat | 3.0 |  |
|  | *ku70^-^* | Lariat | 2.1 | 70 |
|  | *lig4^-^* | Lariat | 2.8 | 93 |
|  | *lig4^-^*; *P{lig4^+^}* | Lariat | 4.0 | 133 |
| 8 | Or-R (WT) | Linear | 0.031 |  |
|  | *polQ^-^* | Linear | <0.0007 | <2 |
|  | Or-R (WT) | Lariat | 0.9 |  |
|  | *polQ^-^* | Lariat | 0.32 | 36 |
| 9 | Or-R (WT) | Linear | 0.019 |  |
|  | *polQ^-^* | Linear | <0.0007 | <4 |
|  | Or-R (WT) | Lariat | 3.1 |  |
|  | *polQ*^-^ | Lariat | 0.54 | 17 |
| 10 | Or-R (WT) | Linear | 0.022 |  |
|  | *polQ^-^* | Linear | 0.00012 | 0.5 |
|  | Or-R (WT) | Lariat | 1.5 |  |
|  | *polQ*^-^ | Lariat | 1.2 | 80 |

Retrohoming assays using lariat and linear RNPs were done in *D. melanogaster* precellular blastoderm embryos, as described in Figure 2A and Materials and Methods. After incubating the embryos for 1 h at 30°C, nucleic acids were extracted and transformed into *E. coli* HMS174(DE3) for plating assays of retrohoming efficiency. WT, wild type.

^a^ Retrohoming efficiency calculated as (Tet^R^ + Amp^R^)/Amp^R^ colonies.

^b^ Retrohoming efficiency relative to wild-type *w^1118^* or Or-R assayed in parallel.
